# Rationalizing the Influence of Small-Molecule Dopants on Guanine Crystal Morphology

**DOI:** 10.1021/acs.chemmater.4c01771

**Published:** 2024-09-01

**Authors:** Avital Wagner, Adam Hill, Tali Lemcoff, Eynav Livne, Noam Avtalion, Nicola Casati, Benson M. Kariuki, Ellen R. Graber, Kenneth D. M. Harris, Aurora J. Cruz-Cabeza, Benjamin A. Palmer

**Affiliations:** † Department of Chemistry, Ben-Gurion University of the Negev, Be’er Sheba 8410501, Israel; ‡ Department of Chemical Engineering, The University of Manchester, Manchester M13 9PL, U.K.; § Department of Chemistry, University of Durham, Lower Mount Joy, South Road, Durham DH1 3LE, U.K.; ∥ Paul Scherrer Institute (PSI), Forschungsstrasse 111, Villigen 5232, Switzerland; ⊥ School of Chemistry, Cardiff University, Cardiff CF10 3AT, Wales, U.K.; # Institute of Soil, Water and Environmental Sciences, The Volcani Institute, Agricultural Research Organization, Rishon Letzion 7528809, Israel

## Abstract

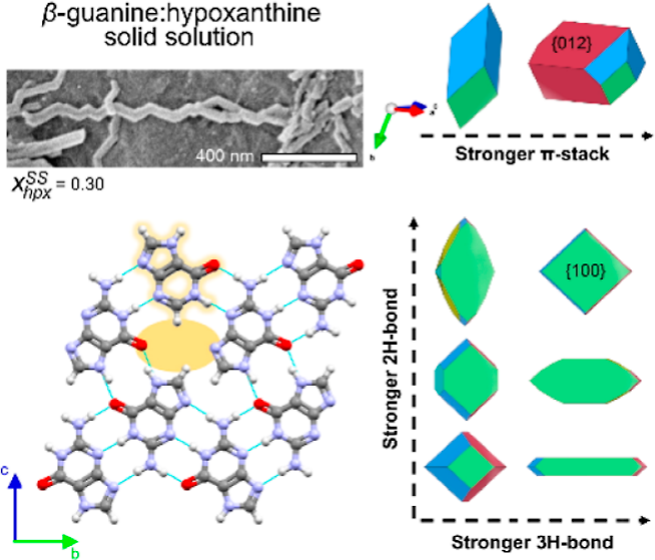

Many spectacular optical phenomena in animals are produced by reflective assemblies of guanine crystals. The crystals comprise planar H-bonded layers of π-stacked molecules with a high in-plane refractive index. By preferentially expressing the highly reflective π-stacked (100) crystal face and controlling its cross-sectional shape, organisms generate a diverse array of photonic superstructures. *How is this precise control over crystal morphology achieved?* Recently, it was found that biogenic guanine crystals are composites, containing high quantities of hypoxanthine and xanthine in a molecular alloy. Here, we crystallized guanine in the presence of these dopants and used computations to rationalize their influence on the crystal morphology and energy. Exceptional quantities of hypoxanthine are incorporated into kinetically favored solid solutions, indicating that fast crystallization kinetics underlies the heterogeneous compositions of biogenic guanine crystals. We find that weakening of H-bonding interactions by additive incorporation elongates guanine crystals along the stacking direction—the opposite morphology of biogenic crystals. However, by modulation of the strength of competing in-plane H-bonding interactions, additive incorporation strongly influences the cross-sectional shape of the crystals. Our results suggest that small-molecule H-bond disrupting additives may be simultaneously employed with π-stack blocking additives to generate reflective platelet crystal morphologies exhibited by organisms.

## Introduction

From the colors of chameleons^1^ and fish^2^ to the mirrored eyes of scallops,^3^ highly reflective assemblies of guanine crystals are responsible for a plethora of optical phenomena in animals.^4−6^ The monoclinic β-guanine crystals are comprised of π-stacked, H-bonded layers,^7^ and exhibit an extremely high refractive index (real part) within the H-bonded plane (*n* = 1.83).^6^ To enhance the reflectance of these systems, organisms produce platelet crystals expressing the highly reflective^4^ but hydrophobic (100) crystal face (parallel to the H-bonded layer)—whose formation is disfavored in aqueous media.^8,9^ Organisms also manipulate the cross-sectional shape of the (100) face to produce square,^3^ hexagonal,^2,10,11^ and irregular polygonal crystals,^12−14^ enabling the assembly of crystals into different photonic superstructures.^4,5^ Rationalizing how organisms precisely regulate crystal morphology to generate different photonic devices is a key goal of *organic biomineralization*, which may inspire new strategies for controlling the morphologies and functions of synthetic molecular materials.

Like inorganic minerals, biogenic guanine crystals are composite materials, containing macromolecular^13,15^ and small-molecule intracrystalline additives.^14,16^ Wagner et al.^13^ discovered that guanine crystals in spiders are intercalated with layers of macromolecules, and similar observations were made in fish^15^ and lizards.^17^ Pinsk et al.^16^ also showed that biogenic guanine crystals are molecular alloys, containing large quantities of hypoxanthine and xanthine dopants which can constitute up to 20 mol % of the crystal. However, the complexity of studying biogenic systems has thus far prevented the rationalization of the effect of these additives on crystal morphology. It is not understood how guanine crystals accommodate such large quantities of small-molecule additives and how these additives influence growth. Previously, it was hypothesized that purine additives may inhibit growth along the π-stacking direction to form plates, parallel to the H-bonded layers.^16,18,19^

**Figure 1:**
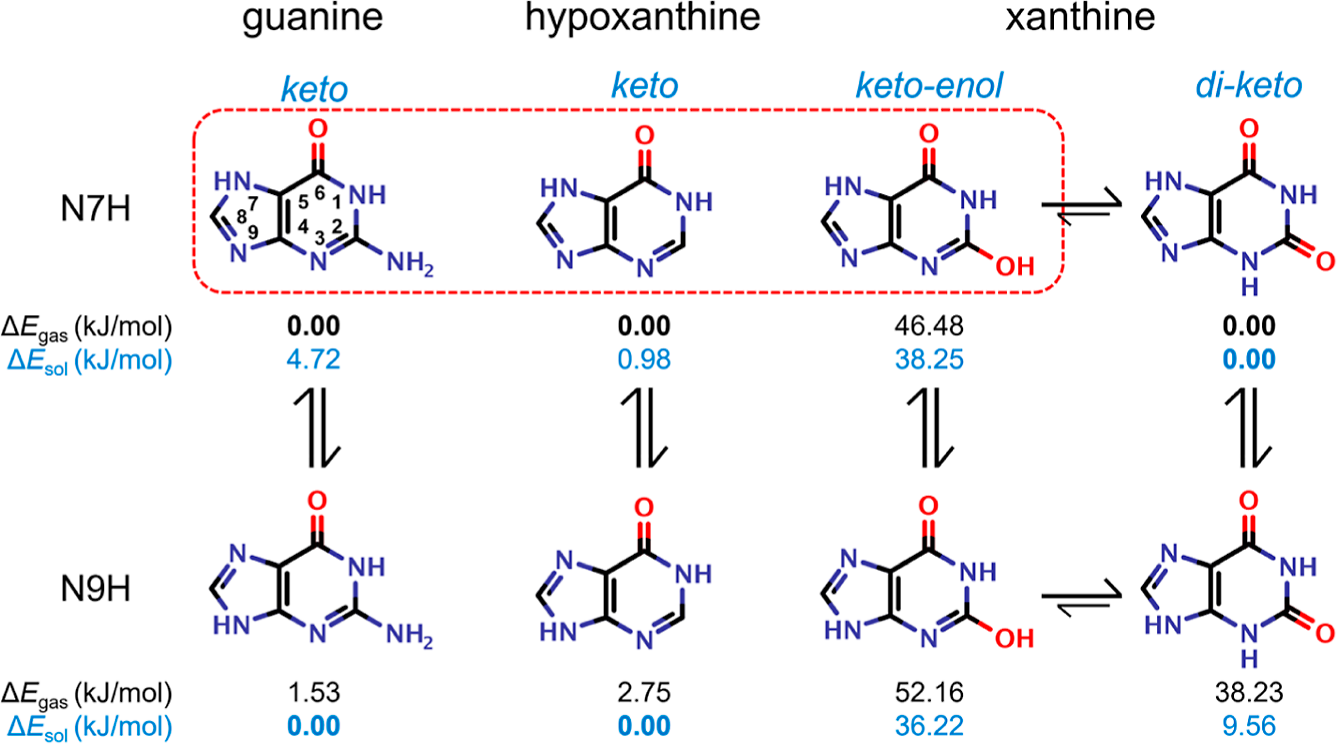
Molecular structures of guanine, hypoxanthine (hpx), and xanthine (xan) exist in different tautomeric forms. DFT-d calculated relative energies of tautomers in the gas phase and solvated in water are shown. Tautomers that have a H-bonding arrangement matching that of guanine in the α/β-guanine crystal structures are contained in the red square.

Here, we crystallized guanine in the presence of hypoxanthine and xanthine (Figure 1) and, together with accompanying calculations, determined: (i) the energetic driving force for solid solution formation, (ii) the influence of additives on guanine morphology, and (iii) the competing intermolecular interactions governing crystal morphology. We find that the formation of metastable guanine solid solutions is favored by fast crystallization or high supersaturation, indicating that such conditions underlie the heterogeneous compositions of biogenic guanine crystals. Counter to previous suggestions, weakening of in-plane H-bonding interactions by additive incorporation elongates the crystals along the stacking direction. However, this disruption of H-bonding interactions also strongly alters the cross-sectional shape of the guanine crystals. Calculations show that by modulating the relative strength of two competing H-bonding interactions, a range of cross-sectional crystal morphologies, resembling biogenic guanine crystals, can be generated. This suggests a novel role for small molecule dopants in crystal morphology regulation in biology.

## Results and Discussion

**Figure 2:**
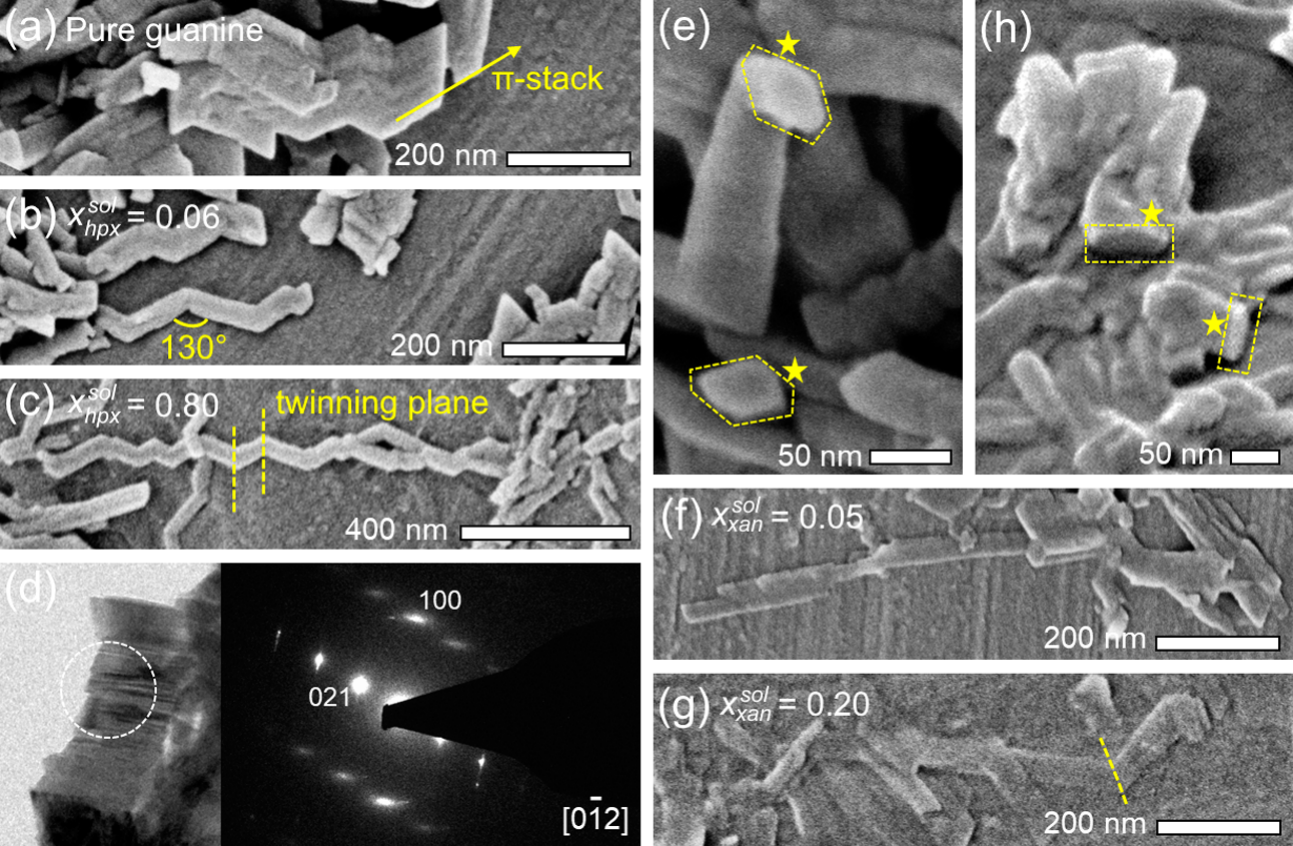
Morphologies of guanine crystals grown in the presence of hpx and xan. (a–c) SEM images of crystals of (a) pure guanine, and guanine grown with a *x*_hpx_^sol^ of (b) 0.06, (c) 0.80. (d) TEM image of a crystal with the corresponding selected area electron diffraction pattern, showing elongation of the crystals along the [100]. (e) SEM image of crystals with a tilted view (45°) showing the pseudohexagonal cross-section of crystals grown with hpx. (f,g) SEM images of crystals obtained at *x*_xan_^sol^ of (f) 0.05 and (g) 0.20. (h) Tilted SEM image of crystals showing the flat cross-section of crystals grown with xan.

To observe how small-molecule additives affect the morphology of guanine crystals, we crystallized guanine in the presence of hypoxanthine (hpx) and xanthine (xan) (experimental and computational details), whose concentrations in solution (relative to guanine) are reported as a molar fraction, *x*_hpx_^sol^ or *x*_xan_^sol^. Guanine and the additive were dissolved in aqueous solutions at pH 13, and crystallization was induced by gradually lowering the pH until precipitation at pH 9 (Figures 2 and S1) or pH 10.5 (Figure S2).^8^ Scanning electron microscopy (SEM) and electron diffraction show that as *x*_hpx_^sol^ increased, the initially prismatic guanine crystals (Figure 2) elongated into needles along the [100] π-stacking direction (Figure 2b–d) and attained a pseudohexagonal cross-section (Figure 2e). All crystals exhibited a “chevron” morphology produced by twinning on the (100) face (Figure 2b–d). The presence of xan also resulted in crystal elongation, with the orientation of the twinning plane demonstrating that the crystals were elongated along the [100] (Figure 2f,g). In contrast to hpx, these crystals became distinctly flattened, exhibiting a lath morphology (Figure 2f–h).

**Figure 3:**
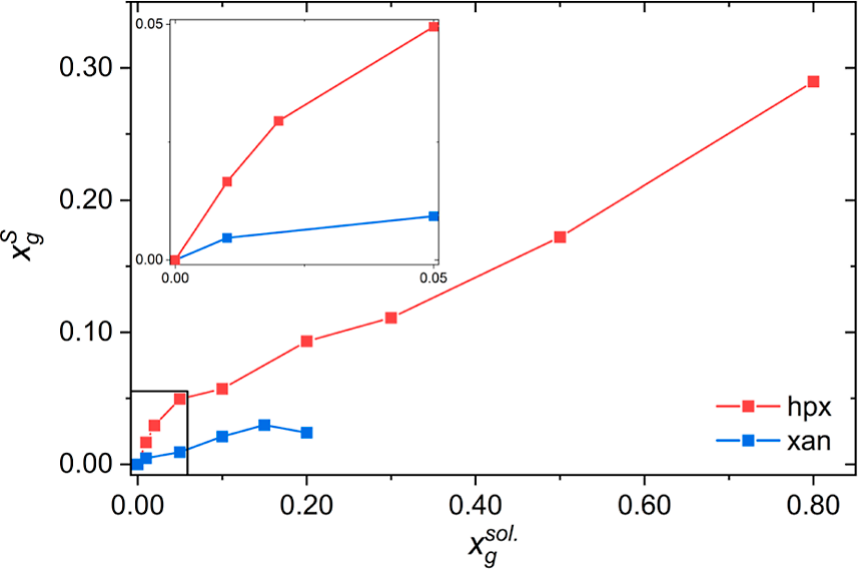
Chemical composition of the crystals determined by HPLC. Plots of *x*_hpx_^S^ (red) and *x*_xan_^S^ (blue) as a function of the molar fraction of guest in solution, *x*_g_^sol^. Inset: an enlarged view of the region below *x*_*g*_^sol^ = 0.05 and *x*_*g*_^S^ = 0.05.

To assess whether changes in the crystal morphology correlate with changes in the crystal composition, high performance liquid chromatography (HPLC) was used to determine the average guanine/additive concentrations of dissolved crystals (Figures 3, S3, and S4, and Table S1). As *x*_hpx_^sol^ increased, the mole fraction of hpx within the solid phase (termed *x*_hpx_^S^) also increased, reaching a maximum of 0.30 at *x*_hpx_^sol^ = 0.8 (Figure 3). In contrast to hpx, only low proportions of xan were included, reaching a maximum incorporation of *x*_xan_^S^ ≅ 0.03 for *x*_xan_^sol^≥ 0.15. Upon increasing *x*_xan_^sol^ from 0 to 0.20 (Figure 3), there was only a slight increase in *x*_xan_^S^, and a similar lath morphology was seen for all crystals (Figure 2f,g), reflecting the minimal changes in the crystal composition in this range. Synchrotron PXRD confirms that hpx and xan form solid solutions within the β-guanine structure (Figures S5–S7). The additive mole fraction of solid solutions is termed *x*_hpx_^SS^ or *x*_xan_^SS^. Changes in the lattice parameters with respect to pure β-guanine (Tables S2 and S3, Figure S7) result from hpx and xan perturbating intermolecular interactions along particular crystallographic directions.

Our results show that large amounts of hpx and smaller amounts of xan are incorporated within synthetic β-guanine solid solutions, which exhibit similar doping levels to biogenic crystals.^16^ Guanine thus acts as an extremely labile “host” system for hpx incorporation, despite its inclusion disrupting the H-bonding network of guanine. At maximum incorporation, more than one molecule out of four in the crystal is hpx.

**Figure 4:**
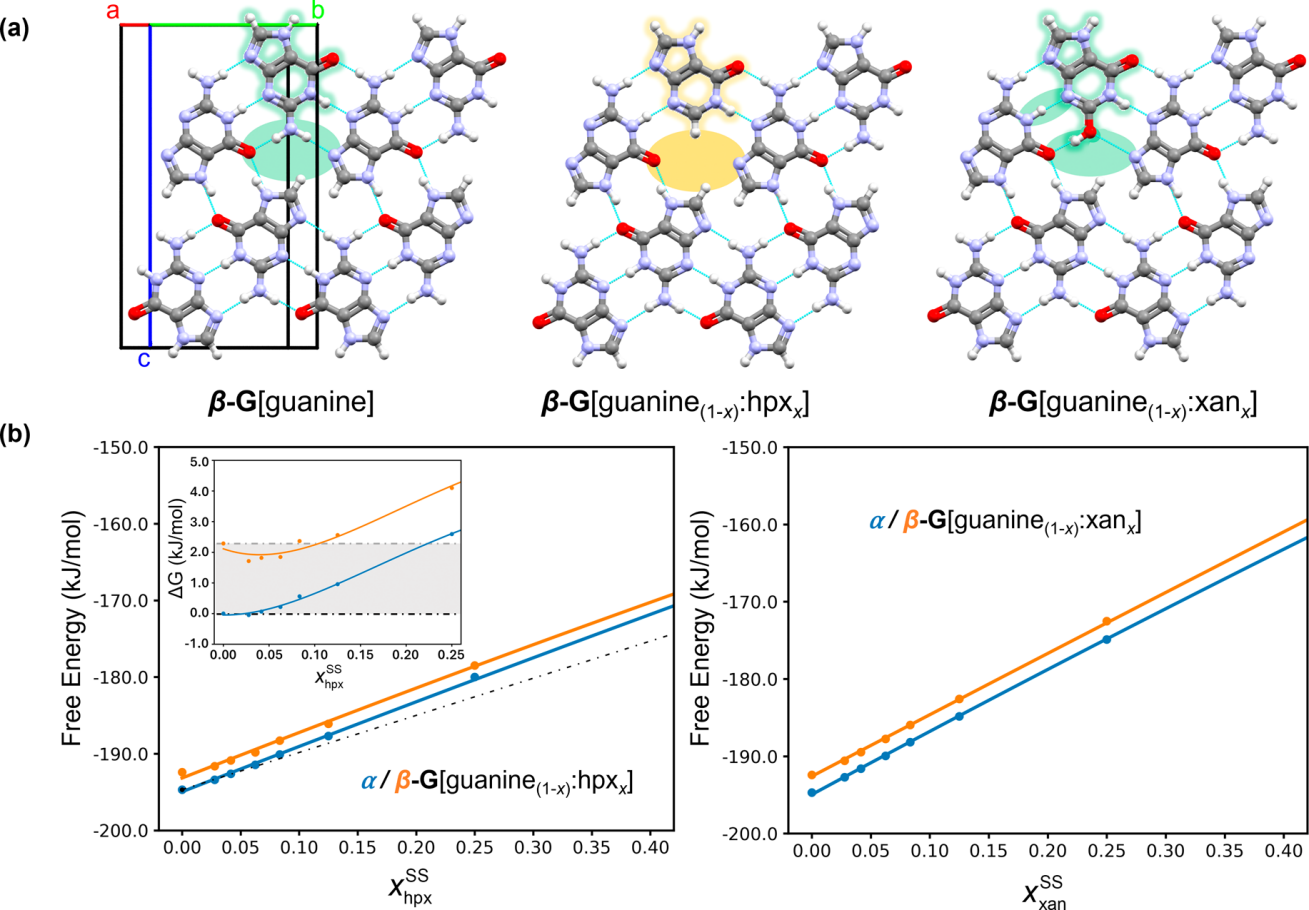
Guanine and solid solution crystal structures and DFT-d energy calculations. (a) Crystal structures of β-guanine, β-guanine-hpx, and β-guanine-xan solid solutions. The orange-green color scale highlights changes to the H-bonding network caused by the additive incorporation, with orange colors representing the highest disruption to the H-bonding network. (b) Free-energy DFT-d calculations of hpx (left) and xan (right) incorporating into α- or β-guanine, assuming ideal solid solutions for the calculation of the entropy of mixing term. Free energies for hpx are plotted alongside the free energy of a physical mixture of α-guanine and α-hpx (dashed line). The inset displays the solid solution energies relative to the energy of the physical mixture. The shaded region illustrates the free energy difference between pure α-and β-guanine.

Typically, such high doping levels in solid solutions are achieved in systems where additives do not perturb strong intermolecular bonding interactions.^20−22^ Thus, to understand the energetic driving force for solid solution formation, we calculated the free energies of α- and β-guanine solid solutions using a DFT-d method recently applied to benzamide crystals^23^ (experimental and computational details). Purines exist in a variety of tautomers (Figure 1) and consideration of these tautomers is crucial for correctly modeling solid solutions (Figures S8 and S9).^24−26^ In both α and β-guanine crystals, the keto-N7H tautomer of guanine is present (Figure 1, top). Calculations were made (Figures S8 and S9), which show that solid solutions of guanine and the keto-N7H tautomer of hpx have lower energies than those with the keto-N9H tautomer due to its favorable H-bonding arrangement with the neighboring keto-N7H guanine (Figure 4a). Similarly, solid solutions of the keto–enol-N7H tautomer of xan have significantly lower energies than those of the diketo-N7H tautomer whose incorporation severely disrupts the H-bonding network of the host (Figures S10 and S11).

Incorporation of either hpx or xan into α or β-guanine results in an increase in the free energies. As the dopant content is increased, the lattice energy of β-guanine-xan solid solutions increases at roughly double the rate of β-guanine-hpx solid solutions (Figure 4b)—consistent with the lower doping levels observed for xan (a maximum of *x*_xan_^S^ = 0.03). α-Guanine solid solutions are more stable than those of β-guanine at all of the compositions studied experimentally. For the hpx system, where a pure crystal structure is known (α-hpx),^27,28^ the free energies of both α- and β-guanine-hpx solid solutions are higher than the free energy of the physical mixture of α-guanine and α-hpx (Figure 4b, inset), showing that hpx solid solutions are thermodynamically metastable. We note, however, that our calculated free energies assume ideal solid solutions for the calculation of the entropy of mixing term. While this is a good approximation when the content of dopant (hpx or xan) within guanine is low, it may deviate at higher contents of dopant. With our model, the free energy of β-guanine-hpx solid solutions always remains within +4 kJ/mol (or 2 × RT at 300 K) of the physical mixture energy of the most stable pure solids, indicating the feasibility to form solid solutions under the kinetically driven, high supersaturation conditions (Figures S12 and S13) used in our experiments. This is consistent with the observation that slower crystallization experiments at pH 10.5 led to less hpx incorporation (Figure S2). Additionally, for *x*_hpx_^SS^ < 0.10, the relative energy of β-guanine-hpx solid solutions drops below the energy difference between pure α- or β-guanine polymorphs (Figure 4b insert, shaded). Given that β-guanine forms readily in biogenic and synthetic systems, this suggests that β-guanine-hpx solid solutions are likely to form when hpx is present in solution. Our results suggest that the heterogeneous composition of biogenic guanine crystals arises spontaneously from the presence of different purine metabolites in the crystallization environment,^16^ coupled with fast crystallization kinetics.

The availability of guanine-compatible hpx and xan tautomers may also explain the preferential incorporation of hpx over xan. The keto-N7H tautomer of hpx, which is compatible with the β-guanine H-bonding network, is also the most stable and abundant hpx tautomer in solution (pH 9). In contrast, the keto–enol-N7H tautomer of xan is the higher energy xan tautomer, which will be significantly less abundant in solution (Figure 1). Moreover, using experimental p*K*_a_ values, the fraction of uncharged guanine, hpx, and xan molecules available for crystallization is 0.71, 0.47, and 0.03 at pH 9, respectively. This suggests that the abundant keto-N7H hpx tautomer will be more readily incorporated than the very scarce keto–enol-N7H xan. This emphasizes that speciation of additives (charged and tautomeric species) is likely to play an important role in their incorporation into β-guanine during crystal growth.^26^

**Figure 5:**
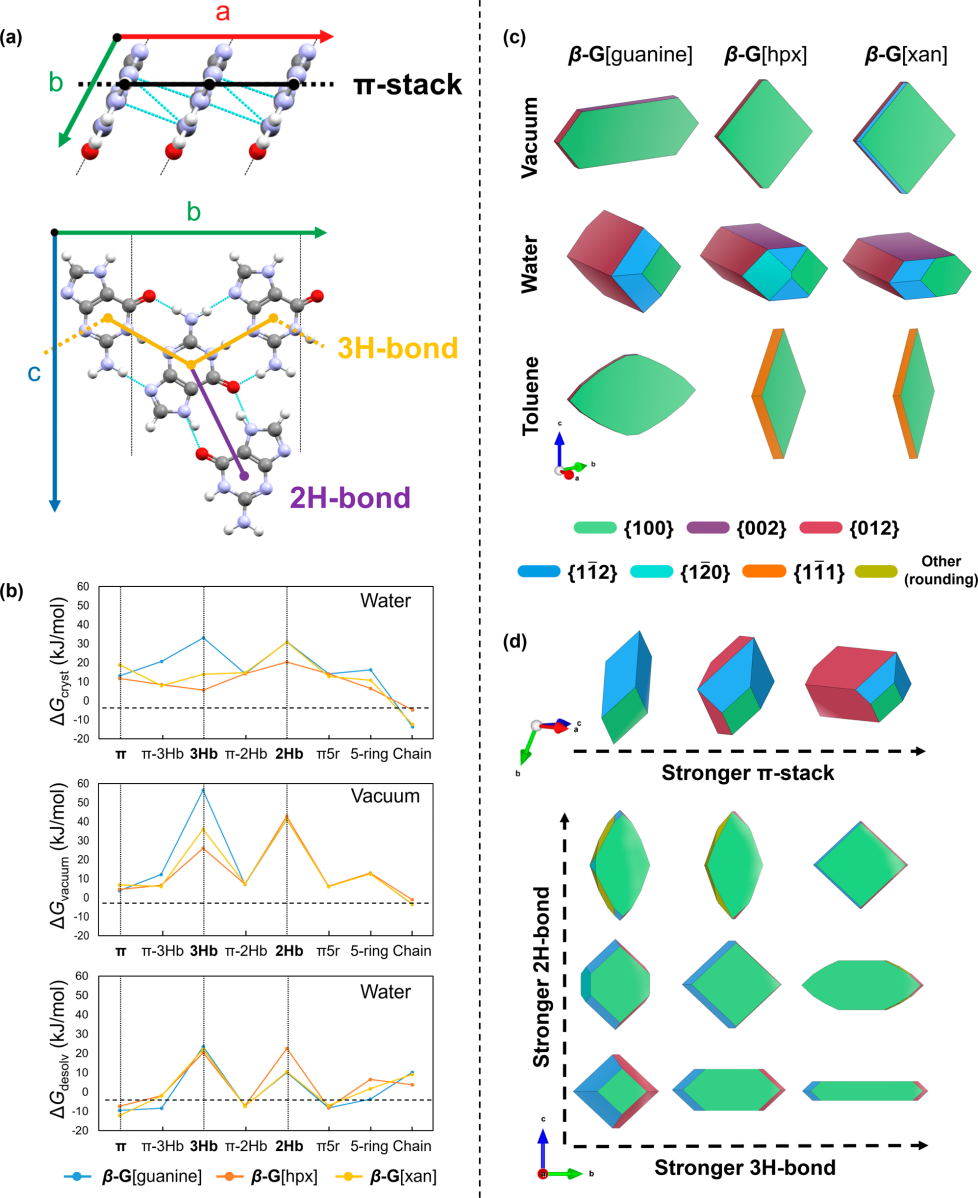
Morphology calculations were performed with **Crystal***Grower*. (a) Three primary interactions (out of the total eight unique interactions) in the β-guanine structure labeled with shorthand notation. (b) Energy components per interaction for β-G[guanine], β-G[hpx], and β-G[xan]. Top; free energy of crystallization (Δ*G*_cryst_) in water, middle; free energy of the solid phase in vacuum (Δ*G*_vacuum_), bottom; and free energy of desolvation in water (Δ*G*_desolv_). Each interaction containing π denotes that it is in the next π-stacked level above or below the central molecule. Equivalent plots of free energies in DMSO and toluene are shown in Figures S19 and S20. (c) Simulated crystal morphologies for pure β-G[guanine], β-G[hpx], and β-G[xan] grown in vacuum, water, and toluene. Facet sets are colored using the scheme shown in the legend. All crystals were simulated in **Crystal***Grower* then reconstructed using VESTA to display smooth facets without surface topography (Figure S14). (d) Effects of increasing the Δ*G*_cryst_ for the π-stack viewed from an offset angle relative to [001] and increasing the Δ*G*_cryst_ for 3H-bond vs 2H-bond on the crystal morphology. Nonvaried interactions are fixed at the calculated Δ*G*_cryst_ values for β-guanine in water. Interaction diagrams were produced with Mercury, and smooth crystal morphologies were replicated from **Crystal***Grower* by using VESTA.

Given that hpx and xan have similar molecular polarizabilities to guanine,^29,30^ it is unlikely that the refractive index of guanine crystals would be significantly altered by their incorporation. This indicates that, from an optical perspective, there would be no “benefit” in organisms *actively* incorporating dopants. The question therefore arises, what function, if any, do additives perform in biogenic systems? To address this question, we investigated how additive incorporation influences the morphologies of β-guanine using **Crystal***Grower*(31,32) simulations, following previously reported methodology.^32^ For simulations of doped crystals, all of the host molecules in the β-guanine structure were replaced with guest molecules (β-G[guest]). This enables morphological comparisons to pure β-guanine by retaining the crystal symmetry (experimental and computational details). Growth simulations were also performed to account for the effect of guest occupancy, which yielded similar results (Movies S1 and S2). For the simulations, a crystal structure and free energies of crystallization for each neighbor interaction in the structure were calculated in vacuum, water, toluene (Figure 5), DMSO, and formamide (Figures S14 and S16 and Movies S3, S4, and S5).

A free energy of crystallization (Δ*G*_cryst_) for an intermolecular interaction is the energetic change required to replace the interaction to a neighbor in the crystalline solid (Δ*G*_vacuum_) with an equivalent interaction to the solvent (Δ*G*_desolv_). Hence, some intermolecular interactions that are initially strong in the crystal structure (e.g., H-bonds) can be outweighed by the high energetic cost of desolvating said interaction (e.g., H-bonds in a polar, protic solvent like water) (Figure S17, schematic).

The Δ*G*_cryst_ for a particular interaction is given by, Δ*G*_cryst_ = Δ*G*_vacuum_ – Δ*G*_desolv_, where Δ*G*_vacuum_ is computed in vacuo (Δ*G*_desolv_ = 0). The values of Δ*G*_cryst_ for each neighbor interaction control the competing growth rates along various crystallographic directions and thus the overall crystal morphology (Figure 5). Eight symmetry–unique interaction types can be derived in β-guanine from 12 neighbors (Figures 5a,b and S18, and Table S4). However, morphology changes are dominated by changes in Δ*G*_cryst_ for the main π-stacking chain interaction along *a* (termed “π-stack”), the triple H-bond chain running along *b* (termed “3H-bond”), and the secondary H-bond which links guanine dimers in the *bc* plane (termed “2H-bond”) (Figure 5a).

When ignoring solvent contributions (Figure 5b, i.e., Δ*G*_cryst_ directly from the solid state, in “vacuum”), pure β-guanine (Figure 5c) adopts an elongated hexagonal plate morphology. As Δ*G*_cryst_ for the 3H-bond is much higher than all other interactions (Figure 5b), the crystal grows rapidly along the *b*-direction, with growth along *c* retarded by the lower Δ*G*_cryst_ of the 2H-bond. Growth in the stacking direction is very slow due to the low Δ*G*_cryst_ for the π-stack, resulting in (100) appearing as the largest facet (Figure 5c). When guanine molecules are replaced with either hpx or xan, there is a dramatic reduction in the Δ*G*_cryst_ for the 3H-bond due to the interruption of the triple H-bond chain such that growth along *b* is reduced to roughly equal that along *c*, resulting in an isotropic plate (Figure 5c).

Solvents affect the crystal morphology by altering the Δ*G*_desolv_ values (Figure 5b, bottom), and thus Δ*G*_cryst_. For β-guanine in water (Figure 5c), there is a significant weakening of Δ*G*_cryst_ for the 3H-bond, due to the high energy cost of desolvating H-bonding interactions in a polar solvent (Figure 5b), causing growth to slow along *b* (Figure 5c). In contrast, Δ*G*_cryst_ for the π-stack is strengthened due to the negative energy of desolvating this hydrophobic interaction (Figure 5b, Δ*G*_desolv_). Preferential growth along the π-stack drives the morphology toward a rod along *a* (Figure 5c), reminiscent of the experimentally observed morphology (Figure 2a). Upon incorporation of hpx, there is a weakening of the 3H-bond in the solid state with respect to the π-stack due to the H-bond network disruption (Figure 5b, Δ*G*_cryst_). This increases the relative growth along the stacking direction such that the (100) facet almost disappears—resembling the needle β-guanine-hpx solid solution morphology observed experimentally (Figure 2b–e). A similar elongation along the π-stack is observed upon xan incorporation (Figure 5c), albeit with a slightly flattened morphology along *c*, as seen experimentally (Figure 2f–h).

Our calculations show how in water weakening of in-plane H-bonding interactions, upon hpx or xan incorporation, elongates the guanine crystals along the orthogonal stacking direction (Figure 5c)—in agreement with experiments (Figure 2). Hpx and xan thus have the opposite effect on morphology that was previously suggested^18,19^—producing elongated needle or lath crystals rather than platelets. In strongly nonpolar toluene (Figures S19 and S20), Δ*G*_cryst_ values in all compounds are similar to those in the solid state, where *bc* plane H-bonding interactions dominate, and the morphologies revert to flat plates (Figure 5c). Δ*G*_cryst_ for the π-stack is disfavored in all compounds as its Δ*G*_desolv_ is maximized in the nonpolar polar solvent such that Δ*G*_cryst_ has a preference to dissolve, resulting in a large, stable (100) face. These results agree with work on biogenic^33^ and synthetic^34−37^ systems, suggesting that a hydrophobic capping layer is required for inhibiting growth along the π-stack in aqueous media (Figures S14 and S21).

Simulations also show that in contrast to pure β-guanine, β-G[hpx] and β-G[xan] crystals have pseudohexagonal cross sections. For β-G[hpx], the change in the cross-sectional shape of the crystals is caused by the weakening of the 2H-bond relative to the 3H-bond due to the enhanced cost of desolvating this interaction compared to pure guanine (Figure 5b, Δ*G*_desolv_). This results in exposure of the (002) face. This suggests that disruption of the in-plane H-bonded network (also evidenced from lattice distortions in the *bc* plane, Figure S7) by dopant incorporation may dictate the cross-sectional shape of guanine—hitherto a morphological mystery of biogenic crystals. These simulations agree with our experimentally obtained morphologies (Figure 2) where hpx and xan solid solutions exhibit pseudo hexagonal and flattened cross sections, respectively. **Crystal***Grower* simulations (Figures 5d and S21) show that by varying the relative strength of in-plane H-bond interactions (along the [020] and [002] directions), a range of cross-sectional guanine shapes can be obtained, including regular and irregular hexagons, squares, and irregular polygons—morphologies displayed in many biogenic systems.^4^

## Conclusions

We rationalized the effect of small-molecule H-bonding disrupting additives on guanine crystal morphology. Such dopants elongate rather than shorten the crystals along the π-stacking direction, indicating that alternative strategies, including the use of hydrophobic macromolecules,^34,35,38^ are required for generating the highly reflective platelet (100) crystals observed biologically. However, we show how purine additives can control the cross-sectional shape of guanine crystals by modulating the strength of in plane H-bonding interactions—an aspect of crystal morphology control that has not been rationalized. Our results raise the hypothesis that organisms may simultaneously employ hydrophobic macromolecular templates (to inhibit π-stack interactions) and small-molecule H-bond inhibitors (to modulate in-plane interactions) to generate an array of different platelet guanine morphologies (Figure S21).^34,35,38^ Finally, our calculations show that β-guanine-hpx solid solutions are metastable relative to α-guanine, but stable relative to β-guanine at low doping levels, thus rationalizing their formation in systems where β-guanine readily forms. The metastability of the solid solutions relative to α-guanine indicates that fast kinetics or high supersaturation conditions underpin the formation of highly doped molecular crystals, ubiquitous in biological systems.

## Experimental and Computational Details

### Guanine Solid Solution Crystallization

For pure and doped guanine crystallization in aqueous solutions, a method similar to the one reported in Gur et al.^8^ was adopted. 20 mg of guanine powder (Sigma-Aldrich) and respective amounts (0–75 mg) of hypoxanthine (Sigma-Aldrich, >99%) or xanthine (Sigma-Aldrich, >99%) were dissolved in 15 mL solution of 0.1 M NaOH (pH 13). Crystallization was induced by titrating the solution with 0.1 M HCl using an autotitrator (TitroLine 7750, SI Analytics) until pH 9 (Figures 2 and S1) or pH 10.5 (Figure S2) was achieved. The rate was set to 0.1 mL per minute. The obtained crystals were collected and cleaned by centrifugation with DDW.^16^ The experiments were replicated three times with similar results. The aqueous solubilities of guanine, hypoxanthine, and xanthine at pH 7 room temperature are 3.87 × 10^–5^, 5.29 × 10^–3^, and 2.45 × 10^–4^ mol/L, respectively.

### Scanning Electron Microscopy

Cleaned crystals were suspended in a small amount of DDW, and 3 μL of this suspension was left to dry on an aluminum SEM stub. The sample was coated with 5 nm Iridium (Quorum Technologies Ltd., Q150T) and imaged using a HRSEM Gemini 300 SEM (Zeiss) by a secondary electron in-lens detector.

### High-Performance Liquid Chromatography

The measurements were carried out with Agilent 1100 Series high performance liquid chromatography (HPLC) equipped with a photodiode array and an Agilent Zorbax SB-Aq column (150 × 3 mm, 3.5 μm). The solvent systems and gradient elution method were mostly based on the method used by Pinsk et al.^16^ The solvents used were ammonium formate buffer, 10 mM, pH 5 (phase A), and methanol (phase B). The gradient elution carried out was: 0–7 min, 0% B; 7–12 min, linear gradient 40% B; 12–15 min, linear gradient 0% B; 15–18 min, hold at 0% B—followed by 2 min post-run using 100% A. The flow rate was 0.7 mL/min, the injected volume was 10 μL, and the column temperature was maintained at 30 °C. Concentrations of purines in each sample were measured by preparing a calibration curve. Stock solutions of guanine (Sigma-Aldrich, >99%), hypoxanthine (Sigma-Aldrich, >99%), and xanthine (Sigma-Aldrich, >99%) were prepared by dissolving 2.000 ± 0.007 mg (Radwag XA 6.4Y.M PLUS Microbalance) of each purine in 1 mL of perchloric acid 70%. The calibration samples were prepared by dilution with ammonium formate buffer (the elution buffer, phase A). The dilution factors were 500, 200, 50, 20, 10, and 5 (Table S1). The molar concentration of each sample was calculated based on the molar mass of each compound. The detection wavelength used for guanine and hypoxanthine was 250 and 270 nm was used for xanthine. The samples were prepared by dissolving approximately 0.5–1 mg of crystals in 0.5 mL of perchloric acid 70% followed by 10× dilution with the ammonium formate elution buffer. Detection of purines present in the samples was based on their retention times and UV–vis absorption spectrum by comparison with the commercial standards. The retention time of hypoxanthine was approximately 14.3 min, xanthine 14.6 min, and guanine 14.9 min. The concentration of each purine in the samples was measured by using the corresponding calibration curve (Figure S3).

### Powder X-ray Diffraction

Powder X-ray diffraction (PXRD) data for samples of pure guanine, xanthine-doped guanine, and hypoxanthine-doped guanine were recorded on the Materials Science beamline at the Swiss Light Source using radiation of wavelength 0.99961 or 0.99965 Å. From visual inspection of the powder XRD data, the structure of all samples was assigned as the β polymorph of guanine, although in some cases, a small amount of the α polymorph of guanine was also present. Determination of the unit cell parameters for each sample was carried out by Pawley fitting of the powder XRD data (in the range 2θ = 3–40°) using the GSAS II program.^39^ In each case, the initial values of the unit cell parameters were taken from those published previously^40^ for the β polymorph of pure guanine. For the samples containing a small amount of the α polymorph of guanine in addition to the major phase of the β polymorph, the Pawley fitting was carried out as a two-phase refinement.

### Computational Methodology—Solid Solution Cell Generation

Solid solution supercells (up to three unit cells along each axis) of α- and β-guanine were generated by substituting a specific number of guanine molecules with hpx or xan. To account for the inherent disorder in these systems, ensembles of different supercells were generated for each crystal structure (α- or β-guanine) at each composition. Solid solution cells were constructed using a Python code with access to the CSD Python API. Crystal structures KEMDOW and KEMDOW01 were used directly from the CSD as initial pure host structures for α- and β-guanine, respectively. Host guanine molecules were then replaced by guest molecules (hypoxanthine, keto-xanthine, and enol-xanthine) through alignment in space resulting in maximum atom overlap, producing a periodic depiction of a solid solution. Larger supercells were produced to obtain low guest concentrations (e.g., *x*_g_^SS^ = 0.027̇ in a 3 × 3 × 1 supercell of guanine) for sampling, while larger guest concentrations (*x*_g_^SS^ > 0.25) were obtained by substituting multiple guanine molecules within single unit cells. Where multiple guanine molecules are replaced, all combinations of neighboring guest molecules were also simulated. Different cell permutations were also sampled (for the *a* and *b* axes), whenever a supercell was generated (i.e., 2 × 1 × 1 and 1 × 2 × 1).

### Computational Methodology—Optimization Procedure

Solid solution lattice energies (*E*_latt,g_^SS^) were computed according to a previously published procedure^20,23^ where the standard method of calculating lattice energies was adapted for multicomponent crystals. DFT-d was used to optimize all crystal structures and molecules, employing VASP (version 5.4.4),^41−44^ with the PBE functional^45^ and PAW pseudopotentials.^46,47^ Three types of dispersion corrections were used when computing the lattice energies: the Grimme-D2 method,^45^ the Tkatchenko–Scheffler (TS) method,^48^ and the many-body dispersion (MBD) energy method^49,50^ (implemented in *k*-space).^51^ Each structural optimization followed the same order: Grimme with unit cell parameters allowed to vary, Grimme with fixed unit cell parameters, TS with unit cell parameters allowed to vary, TS with fixed unit cell parameters, and finally a single-point calculation of MBD. The total free energies were calculated by adding vibrational contributions and an entropy of mixing term to the computed lattice energies. Since crystal doping does not result in a significant pressure or volume change (PΔ*V* = 0), we assume the Helmholtz and Gibbs energies are interchangeable and use the term G for free energy. Unit cell parameters were extracted from the optimized supercells, and their relative changes with *x*_hpx_^SS^ or *x*_xan_^SS^ agree well with the experimental trends (Figure S7).

In addition to computing the energies for solids, a gas-phase reference value to both the host and guest molecules present within the compound is required to compute *E*_latt,g_^SS^. This is computed by placing a host molecule or guest molecule in a fixed 20 × 20 × 20 Å supercell with the same simulation parameters (i.e., dispersion corrections) as the related crystal structure and optimized. *E*_latt,g_^SS^ for a solid solution at a defined incorporated guest concentration (*x*_g_^SS^) can be calculated with the following equation
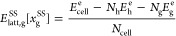
where *N*_cell_, *N*_h_, and *N*_g_ denote the total number of molecules, the number of host (guanine) molecules, and the number of guest (hypoxanthine or xanthine) molecules present in the supercell, respectively, and *E*_cell_^e^, *E*_h_^e^, and *E*_g_^e^ denote the electronic energy of the SS supercell along with the reference energy values for the host and guest in the gas phase, respectively.

As solid solutions are multicomponent solids, the effect of mixing entropy (*ΔS*_mix_^SS^) must also be considered, assuming ideal mixing, this can be computed simply with
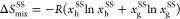
where *x*_h_^SS^ and *x*_g_^SS^ denote the mole fractions of the host and guest compound incorporated within the solid solution, respectively, and R denotes the universal gas constant.

### Vibrational Mode Calculations

To compute the effects of temperature on the stability of solid solutions, the Phonopy^52,53^ tool was employed to calculate vibrational contributions to the free energy via phonon dispersion. Structures optimized to the level of TS with fixed cell parameters were used as inputs for Phonopy processing. Displaced supercells were generated for both α and β-guanine solid solutions with *x*_g_^SS^ values of 0, 0.25, 0.50, 0.75, and 1.0, as calculations for the lower *x*_g_^SS^ values were prohibitively expensive. Supercells of 3 × 1 × 1 and 3 × 2 × 1 were used for α and β-guanine solid solutions, respectively, to obtain unit cell lengths of at least 10 Å as recommended in the literature.^54,55^ The calculations were limited to only keto-hypoxanthine and enol-xanthine as guests as these molecules resulted in the lowest set of lattice energies for the solid solutions at 0 K. Forces were computed using the VASP interface in Phonopy, and the vibrational contribution to the free energy was extracted at 300 K, relative to 0 K. A trendline was plotted for the simulated values using the Python Numpy library polyfit command (*n* = 3) to obtain a value for the vibrational contribution to the free energy across the entire *x*_g_^SS^ range. The value at each respective *x*_g_^SS^ corresponding to a simulated solid solution structure was added to the *E*_latt,g_^SS^[*x*_g_^SS^] to obtain a free energy at each sampled *x*_g_^SS^ (*G*_latt,g_^SS^[*x*_g_^SS^]).

### Physical Mixture Energy Calculations

To confirm whether the solid solution is thermodynamically stable, its energy value must be compared with that of the two most stable components in the system. For guanine + hypoxanthine, this would be the α polymorph of guanine and the α polymorph of hypoxanthine. Xanthine has no known crystal structure for xanthine; therefore, a similar comparison was not shown. The physical mixture energy was computed by assuming a linear relationship between *E*_latt_ of α guanine and α hypoxanthine. Pure *E*_latt_ values were calculated by using the same methodology and parameters as the solid solution cells (including vibrational contributions computed for the pure structures). Physical mixtures of solid solutions can be computed in the same manner, by linking energy values at specific *x*_g_^SS^ values with straight lines and then extrapolating to the energy of the nearest pure component.

### Morphology Calculations

Simulated crystal morphologies were all calculated using **Crystal***Grower* (version X-1.8).^31,32^ Unoptimized CIFs of β-guanine (KEMDOW01 from the CSD) and β-guanine:guest structures were used as the initial crystal structure for morphology modeling. The β-guanine:guest structures were generated with the same approach as the solid solution structures, but here all host molecules in the β-guanine structure were replaced with guest molecules (denoted as β-G[guest]). This enables morphological comparisons to pure β-guanine by retaining the crystal symmetry and avoids excessive computational demands of simulating exponentially increasing numbers of structural combinations at high guest occupancy. Growth simulations of β-guanine:hpx and β-guanine:xan solid solutions were also performed accounting for the effect of guest occupancy (*x*_g_^SS^), which yielded similar results (Movies SM1 and SM2). An exception—the structure containing diketo-xanthine requiredoptimization with fixed unit cell parameters (using the same parameters as the solid solution optimization process with Grimme-D2 corrections) as some hydrogen atoms were too close in space, causing neighboring xanthine molecules to be incorrectly classed as bonded.

Crystals were grown initially with a high supersaturation (>100 kJ/mol) to overcome nucleation barriers and then allowed to equilibrate for the final half of the simulation. Crystals were simulated growing under vacuum, DMSO, water, toluene and formamide using the same parameters aside from the free energies of crystallization for each neighbor interaction, which differ based on the solvent the crystal is growing in. Free energies of crystallization were computed using the Open Computational Chemistry (OCC) library and accompanying program occ-cg,^56^ developed as an interface for **Crystal***Grower*. Single crystals were grown for 1 million growth/dissolution cycles, with a starting thermodynamic driving force of 100 kcal/mol, starting to descend to equilibrium at 500,000 iterations, and reaching equilibrium at 600,000 iterations. Facets were identified using the *d*-spacing coloring method in **Crystal***Grower*. Growth movies were computed over 5 million iterations and composed of 100 frames taken at each 50,000-iteration interval. Smooth facet images were generated using VESTA,^57^ while detailed facet images and movies were generated using OVITO.^58^

### Calculation of Free Energies of Crystallization

Free energies of crystallization were computed as inputs for **Crystal***Grower* with the OCC library using the same CIFs used as inputs from the morphology modeling. This software employs the CrystalExplorer (CE-B3LYP) model^59,60^ and the SMD solvent continuum model^61^ and partitions the solvent surface around a central molecule into sections corresponding to individual dimer/neighbor interactions found within 3.8 Å of a central molecule in the crystal structure. Intermolecular interaction energies are computed for molecules neighboring the central molecule (summing to the lattice energy), and then the free energy of solvation is computed with the SMD model. This computed solvation field is then partitioned according to the neighbors, and the energy cost for desolvating each solvent partition from the central molecule is computed (Figure S15). This partial solvation energy is subtracted from the neighbor interaction energy to result in the free energy of crystallization for each neighbor interaction in a particular model solvent.

Free energies of crystallization in a vacuum were computed using CrystalExplorer, where the same energy model is employed in the absence of solvent. A molecular cluster within 3.8 Å of a central molecule was produced; then, the intermolecular interactions were computed using the “Accurate B3LYP/6-31G(d,p)” setting. Total energies for each interaction were taken from the CrystalExplorer output and divided by two as half bonds need to be considered in **Crystal***Grower*.

## Supporting Information Available

Movie SM1: Simulations of β-guanine-hpx solid solutions with the 3H-bond strength varied between guanine and guest (MP4)
Movie SM2: Simulations of β-guanine-xan solid solutions in water, with 3H-bond strength varied between guanine and guest to control guest incorporation (MP4)
Movie SM3: Timelapse of crystals (pure β-guanine) grown in water (MP4)
Movie SM4: Timelapse of β-G[hpx] grown in water (MP4)
Movie SM5: Timelapse of β-G[xan] grown in water (MP4)
Experimental results and computational results (PDF)
