# Supplementary material for: Rationalizing the Influence of Small-Molecule Dopants on Guanine Crystal Morphology
Source: Chem Mater. 2024 Sep 1;36(18):8910–9. doi: 10.1021/acs.chemmater.4c01771 (PMC11428123; doi:10.1021/acs.chemmater.4c01771)
Supplement: Supplementary file 6 — cm4c01771_si_006.pdf [file cm4c01771_si_006.pdf]

Supplementary Information

Rationalizing the Influence of Small Molecule Dopants on Guanine Crystal Morphology

Avital Wagner,[1†] Adam Hill,[2,3†] Tali Lemcoff,[1] Eynav Livne,[1] Noam Avtalion,[1] Nicola Casati,[4] Benson M. Kariuki,[5] Ellen R. Graber,[6] Kenneth D.M. Harris,[5] Aurora J. Cruz-Cabeza,[3]* Benjamin A. Palmer.[1]*

[1]Department of Chemistry, Ben-Gurion University of the Negev, Be'er Sheba, 8410501, Israel.
[2]Department of Chemical Engineering, The University of Manchester, Manchester, M13 9PL, U.K.
[3]Department of Chemistry, University of Durham, Lower Mount Joy, South Rd, Durham, DH1 3LE, U.K.
[4]Paul Scherrer Institute (PSI), Forschungsstrasse 111, Villigen 5232, Switzerland.
[5]School of Chemistry, Cardiff University, Cardiff CF10 3AT, Wales, United Kingdom.
[6]Institute of Soil, Water and Environmental Sciences, The Volcani Institute, Agricultural Research Organization, Rishon Letzion 7528809, Israel.

†Authors contributed equally.
*Corresponding authors: aurora.j.cruz-cabeza@durham.ac.uk, bpalmer@bgu.ac.il

S1. Supplementary experimental results

Table S1. Dilution factor and concentrations of calibration samples

Sample number	Dilution factor	Concentration (mg/mL)	Concentration (mol/L) guanine	Concentration (mol/L) hpx	Concentration (mol/L) xan
1	500	0.0004	2.65E-06	2.94E-06	2.63E-06
2	200	0.001	6.62E-06	7.35E-06	6.57E-06
3	50	0.004	2.65E-05	2.94E-05	2.63E-05
4	20	0.01	6.62E-05	7.35E-05	6.57E-05
5	10	0.02	0.000132	0.000147	0.000131
6	5	0.04	0.000265	0.000294	0.000263

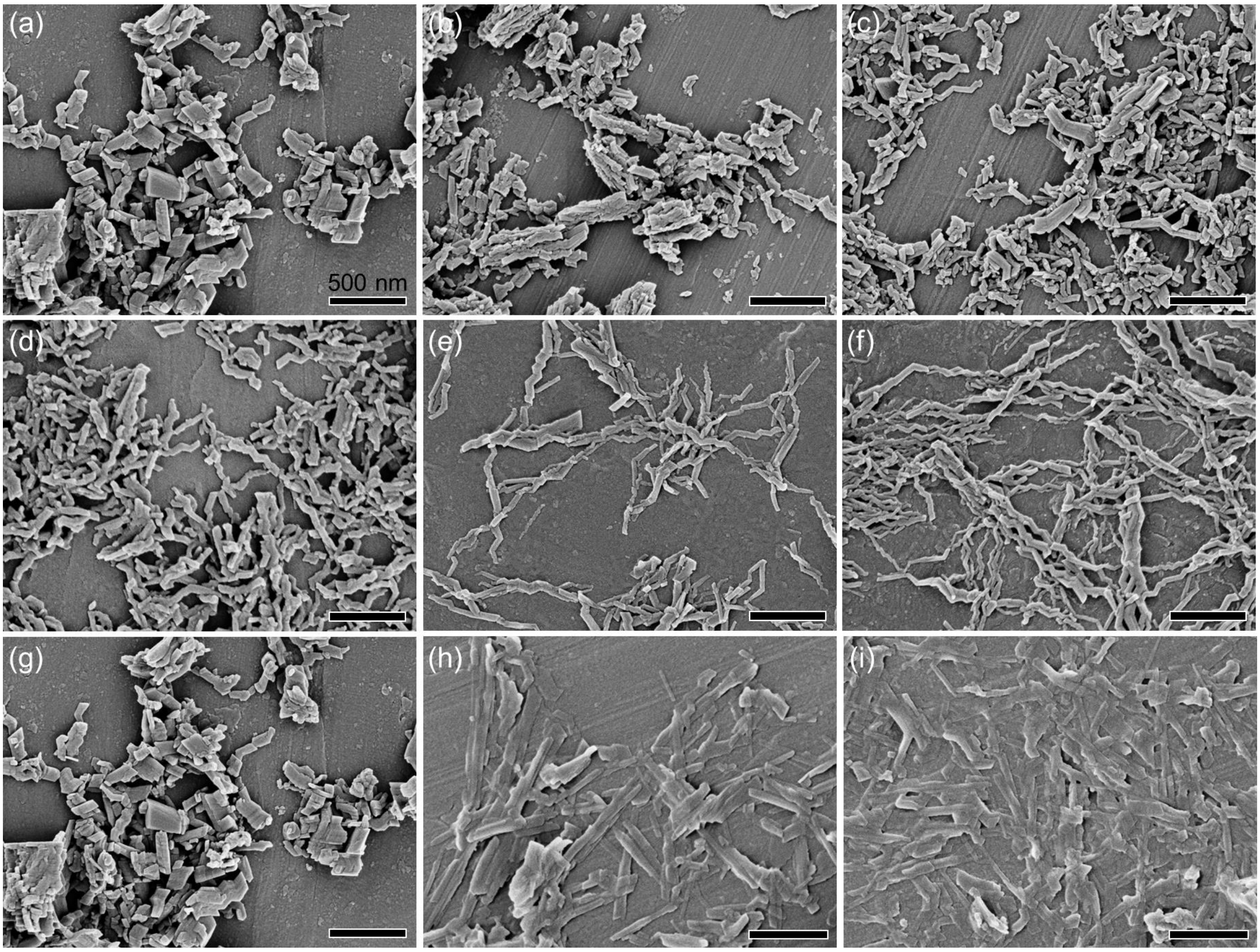

Figure S1. Morphologies of guanine crystals grown in the presence of hpx and xan. (a-f) SEM images of crystals of (a) pure guanine, and guanine grown with a $x_{\mathrm{hpx}}^{\mathrm{sol}}$ of (b) 0.03, (c) 0.06, (d) 0.10, (e) 0.50, (f) 0.80. (g-i) SEM images of crystals of (g) pure guanine, and guanine grown with $x_{\mathrm{xan}}^{\mathrm{sol}}$ of (h) 0.05 and (i) 0.20.

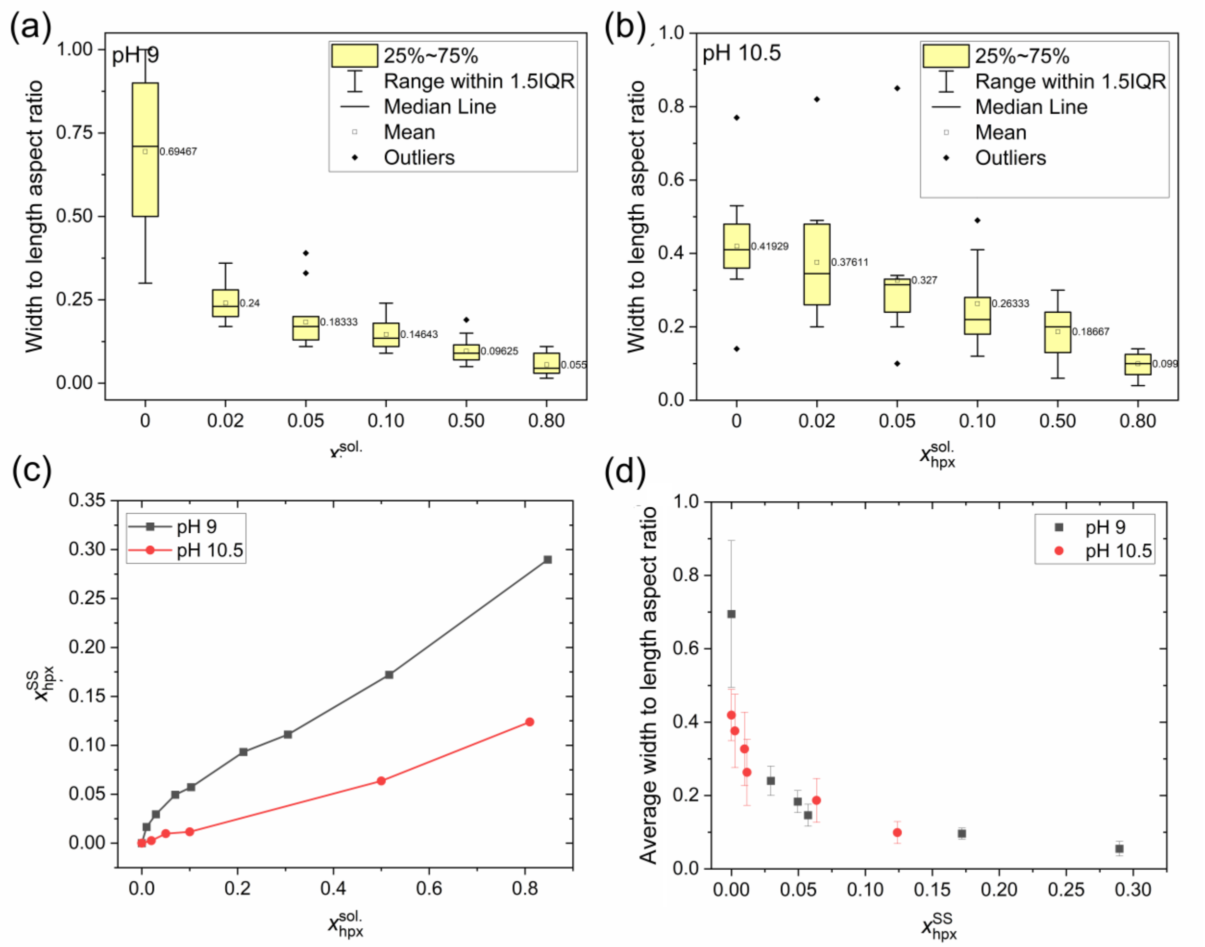

Figure S2. Box plots of the width to length aspect ratio of the *β*-guanine-hpx solid solutions crystallized at (a) pH 9 and (b) pH 10.5 plotted as a function of $x_{\mathrm{hpx}}^{\mathrm{sol.}}$. (c) the $x_{\mathrm{hpx}}^{\mathrm{SS}}$ for each of the pHs used, showing less hypoxanthine was incorporated in pH 10.5 for the same $x_{\mathrm{hpx}}^{\mathrm{sol.}}$. (d) Average width to length aspect ratio for all *β*-guanine-hpx solid solutions showing the effect of hypoxanthine incorporation on the average width to length ratio.

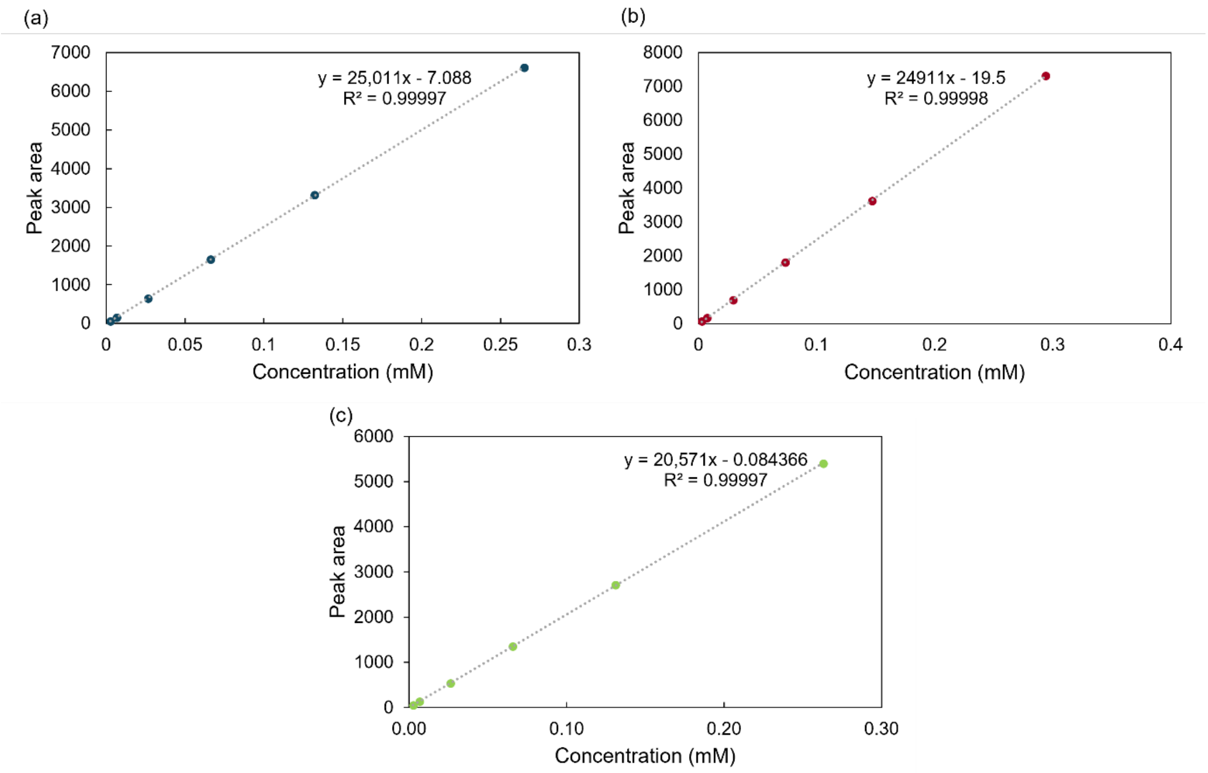

Figure S3. (a) Guanine concentration calibration curve using a 250 nm detection wavelength. (b) Hypoxanthine concentration calibration curve using a 250 nm detection wavelength. (c) Xanthine concentration calibration curve using a 270 nm detection wavelength.

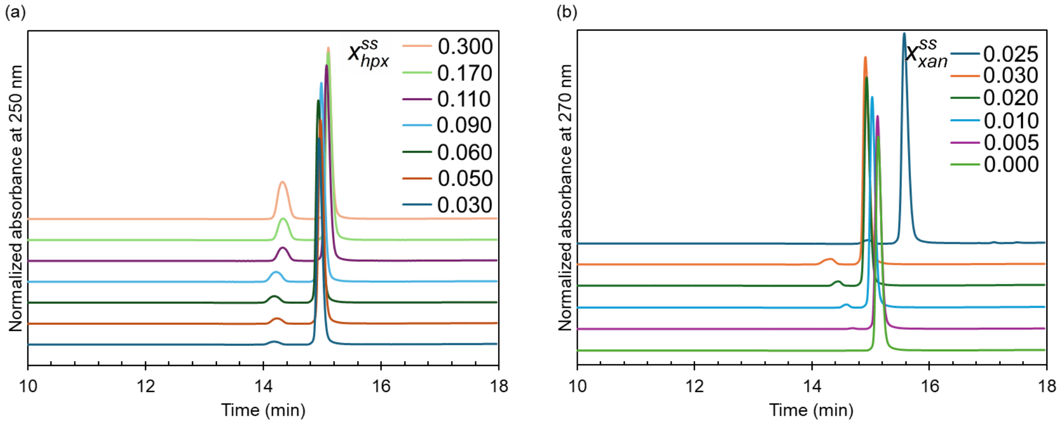

Figure S4. (a) Chromatograms of β-guanine-hpx solid solutions prepared with increasing percentages of initial hypoxanthine. Hypoxanthine retention time ~14.3 min, guanine retention time ~14.9 min. (b) Chromatograms of β-guanine-xan solid solutions prepared with increasing percentages of initial xanthine. Xanthine retention time ~14.6 min, guanine retention time ~14.9 min. The peak intensities were normalized by dividing by the sum of the maximum intensities of the two peaks in each chromatogram.

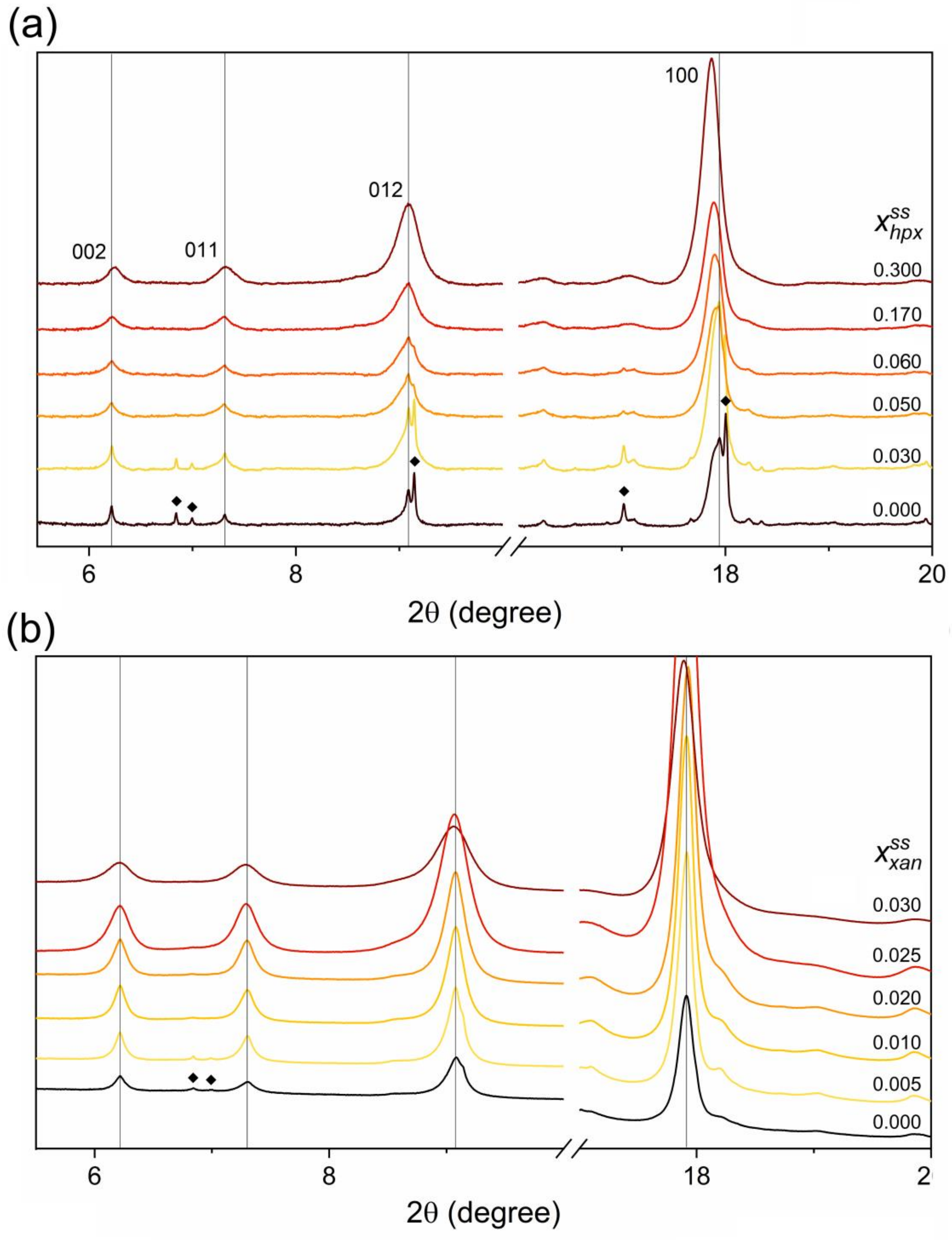

Figure S5. High-resolution synchrotron PXRD data (λ ≈ 1 Å) recorded (a) for samples with increasing $x_{\mathrm{hpx}}^{\mathrm{SS}}$, and (b) for samples with increasing $x_{\mathrm{xan}}^{\mathrm{SS}}$. For samples containing a minor component the α polymorph of guanine together with the major phase of the β polymorph, the peaks due to the α polymorph are indicated by asterisks.

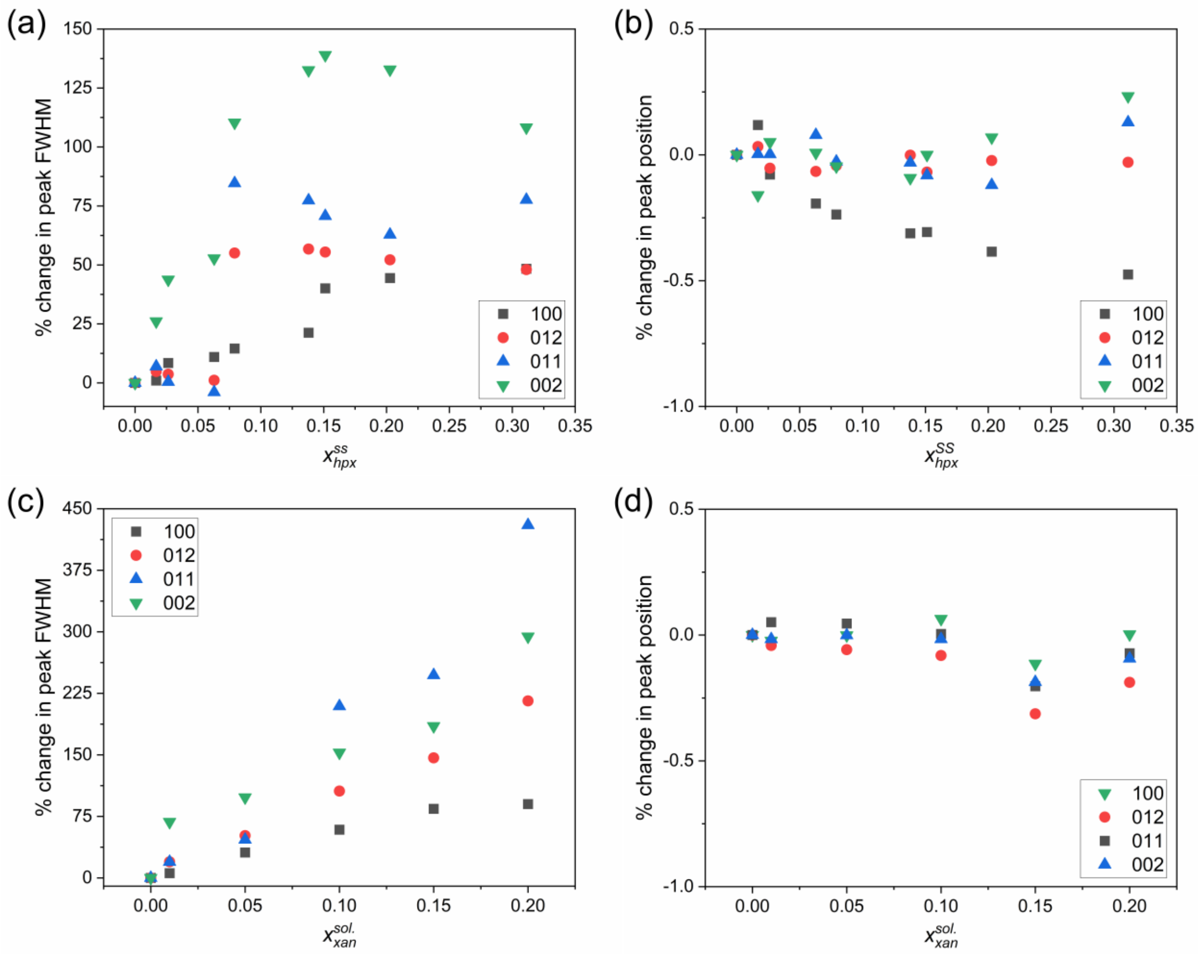

Figure S6. Analysis of PXRD data presented in Figure S5. Percent change in peak (a,c) full width half maximum (FWHM) and (b,d) position from PXRD data for the four main peaks (100), (012), (011), and (002): (a,b) hypoxanthine and (c,d) xanthine.

Table S2. Unit cell parameters and unit cell volume of β-guanine-hpx samples in space group P2$_1$/c determined from Pawley fitting of the powder XRD data.

$x_{\mathrm{hpx}}^{\mathrm{SS}}$	*a* [Å]	*b* [Å]	*c* [Å]	β [°]	Volume [Å^3]
0	3.63195(11)	18.42274(15)	9.81447(71)	117.800(1)	580.895(12)
0.0166	3.6320(21)	18.42372(24)	9.81444(147)	117.798(1)	580.942(15)
0.0295	3.63246(25)	18.42616(26)	9.81834(187)	117.808(1)	581.273(16)
0.0495	3.63267(49)	18.42611(46)	9.81617(36)	117.778(3)	581.128(22)
0.0572	3.63386(34)	18.42538(40)	9.81715(261)	117.785(2)	581.320(22)
0.0932	3.63008(35)	18.41555(49)	9.8155(268)	117.635(2)	581.103(29)
0.111	3.63071(65)	18.41236(72)	9.81607(512)	117.655(4)	581.034(30)
0.1721	3.63241(29)	18.40759(45)	9.8146(21)	117.626(2)	581.222(25)
0.2897	3.63388(42)	18.40184(47)	9.82821(328)	117.603(2)	582.201(25)

Table S3. Unit cell parameters and unit cell volume of β-guanine-xan samples in space group P2$_1$/c determined from Pawley fitting of the powder XRD data.

$x_{\mathrm{xan}}^{\mathrm{SS}}$	a [Å]	b [Å]	c [Å]	β [°]	Volume [Å^3]
0	3.63214(12)	18.42379(16)	9.81495(76)	117.849(1)	580.724(14)
0.0047	3.63301(18)	18.42622(21)	9.81716(121)	117.891(1)	580.849(15)
0.0093	3.63276(17)	18.42696(20)	9.81647(109)	117.886(1)	580.815(17)
0.0211	3.63273(20)	18.43087(22)	9.81531(133)	117.950(1)	580.522(17)
0.0298	3.63132(33)	18.43169(34)	9.81264(261)	117.799(2)	580.973(19)
0.024	3.63158(37)	18.42683(36)	9.81939(263)	117.870(2)	580.884(27)

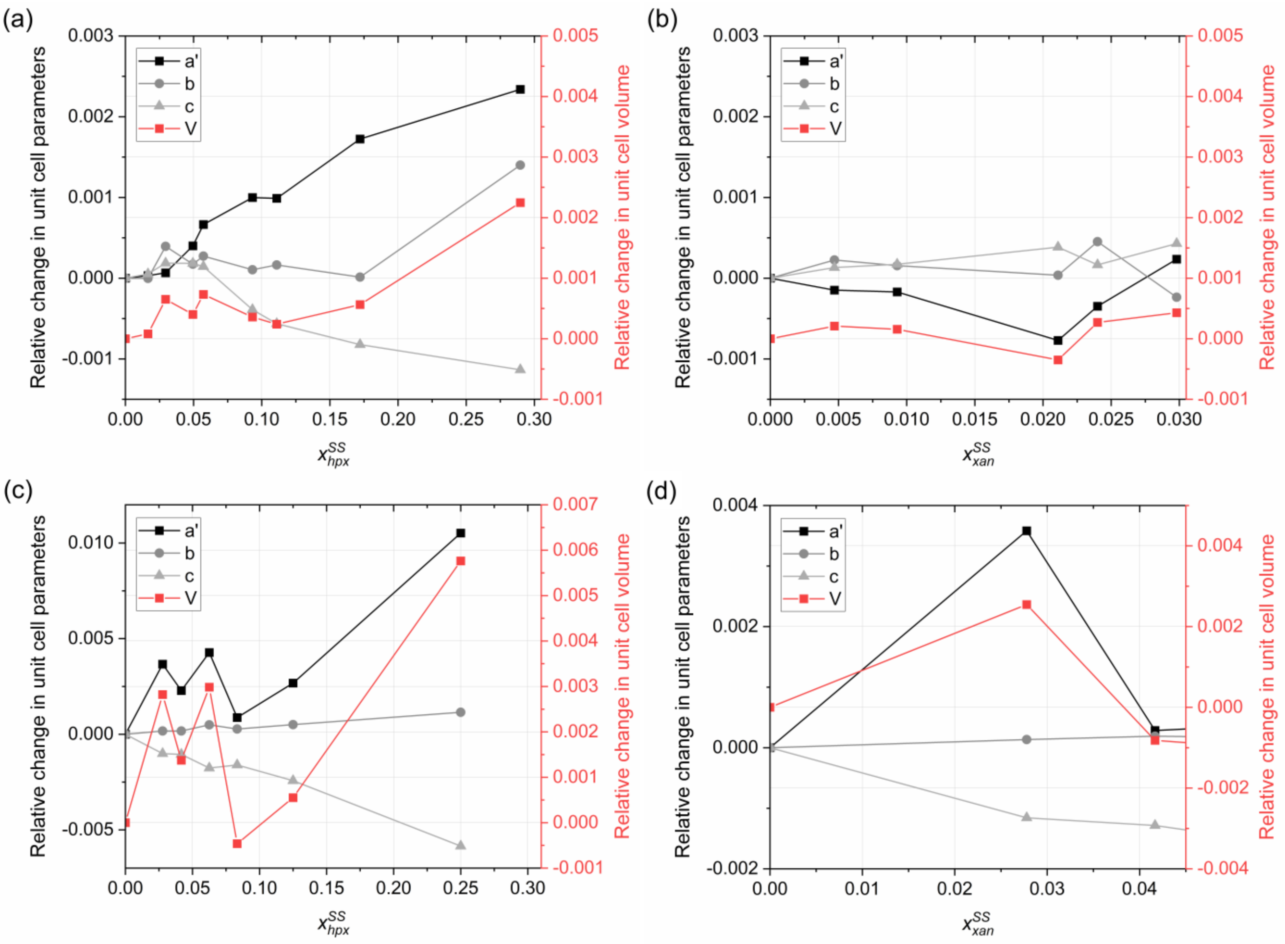

Figure S7. Relative changes in (a,b) the experimental unit cell parameters and (c,d) the computationally determined unit cell parameters as a function of $x_{\mathrm{hpx}}^{\mathrm{SS}}$ or $x_{\mathrm{xan}}^{\mathrm{SS}}$ for the ranges of $x_{\mathrm{hpx}}^{\mathrm{SS}}$ or $x_{\mathrm{xan}}^{\mathrm{SS}}$ in the experimentally prepared samples. Relative changes in unit cell volume are displayed in red. The perpendicular distance between H-bonded layers is denoted a′, and is given by: $a' = a\cos(\beta - 90°)$. The computed unit cell parameters are those obtained following the full optimization procedure (Grimme-D2 > TS > MBD). There is relatively good agreement between the trends observed in the experimental and computational results. For the β-guanine:hpx solid solutions, increasing $x_{\mathrm{hpx}}^{\mathrm{SS}}$ is associated with expansion of the unit cell along the π-stacking direction, suggesting a weakening of the stacking interactions (Tables S2-S3). Increasing $x_{\mathrm{hpx}}^{\mathrm{SS}}$ is also associated with contraction along the c-axis and expansion along the b-axis. These observations indicate that the β-guanine structure is also perturbed in the H-bonded plane by additive inclusion, although to a lesser extent than the perturbations along the π-stacking direction. For the β-guanine:xan solid solutions, increasing $x_{\mathrm{xan}}^{\mathrm{SS}}$ is associated

with minimal changes in the unit cell parameters and unit cell volume, consistent with the lower doping levels observed in this system.

S2. Supplementary computational results

The solid solution system is referred to as β-G[guanine$_{(1-x)}$:guest$_x$], where 'β-G' is the guanine crystal structure with its composition in brackets.

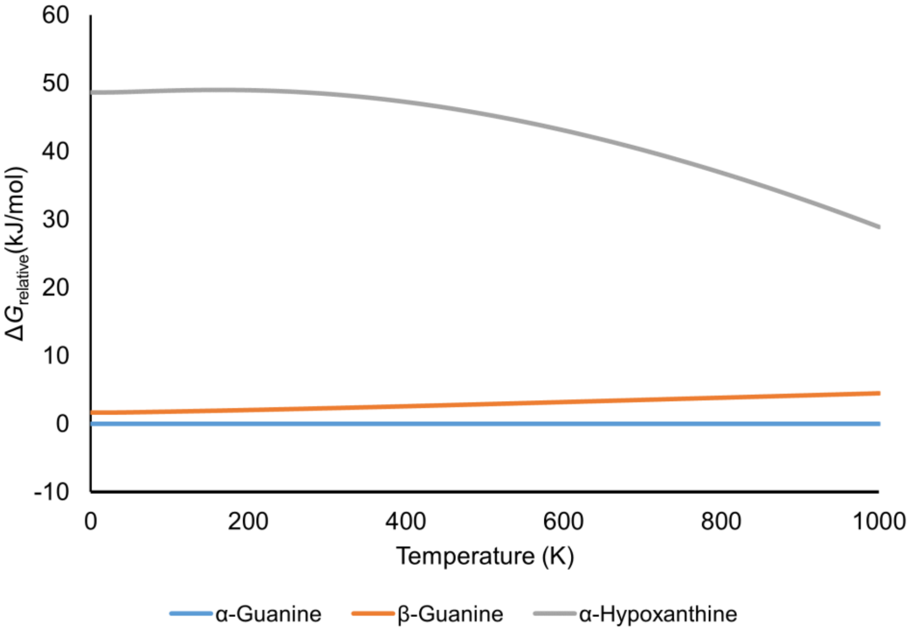

Figure S8. Relative free energies ($\Delta G_{\text{relative}}$) of pure α-guanine, β-guanine and α-hypoxanthine relative to the free energy of α-guanine with increasing temperature (calculated using VASP and Phonopy).

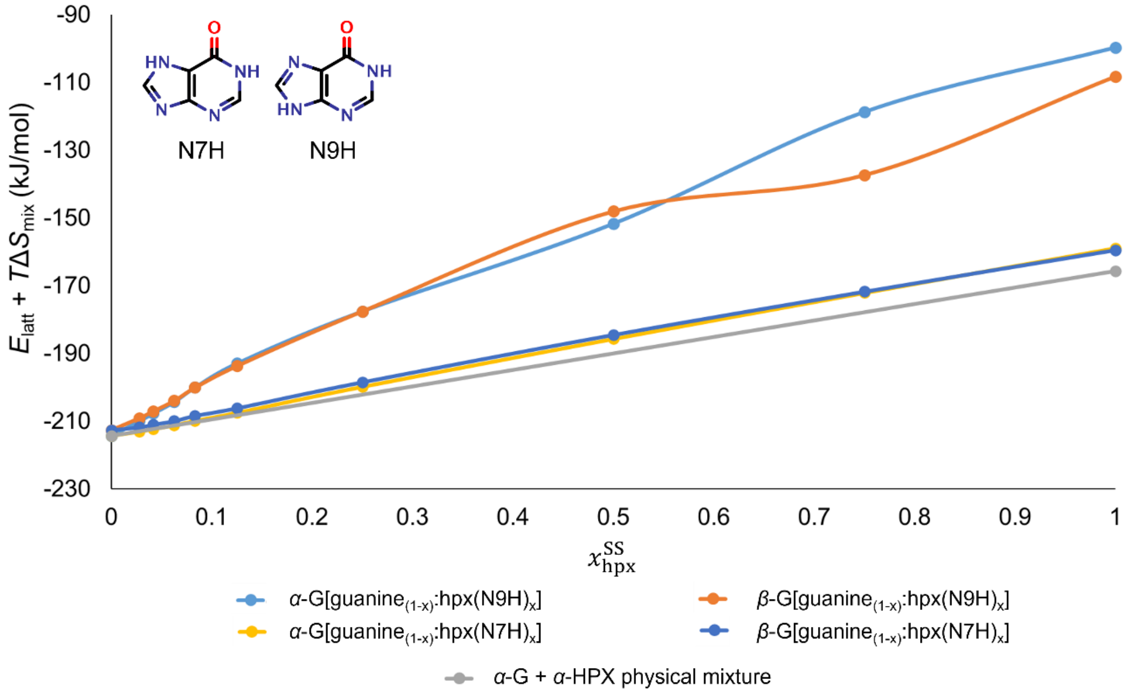

Figure S9. Lattice energy plots for all tautomers of hypoxanthine with added entropic corrections to account for solid solution formation (TS + MBD corrections at 0 K). The N9H tautomer causes energies to increase dramatically when incorporating into either guanine polymorph, due to the mismatch in the hydrogen position causing steric clashing. The N7H position is easier to accommodate, given its similarity to guanine.

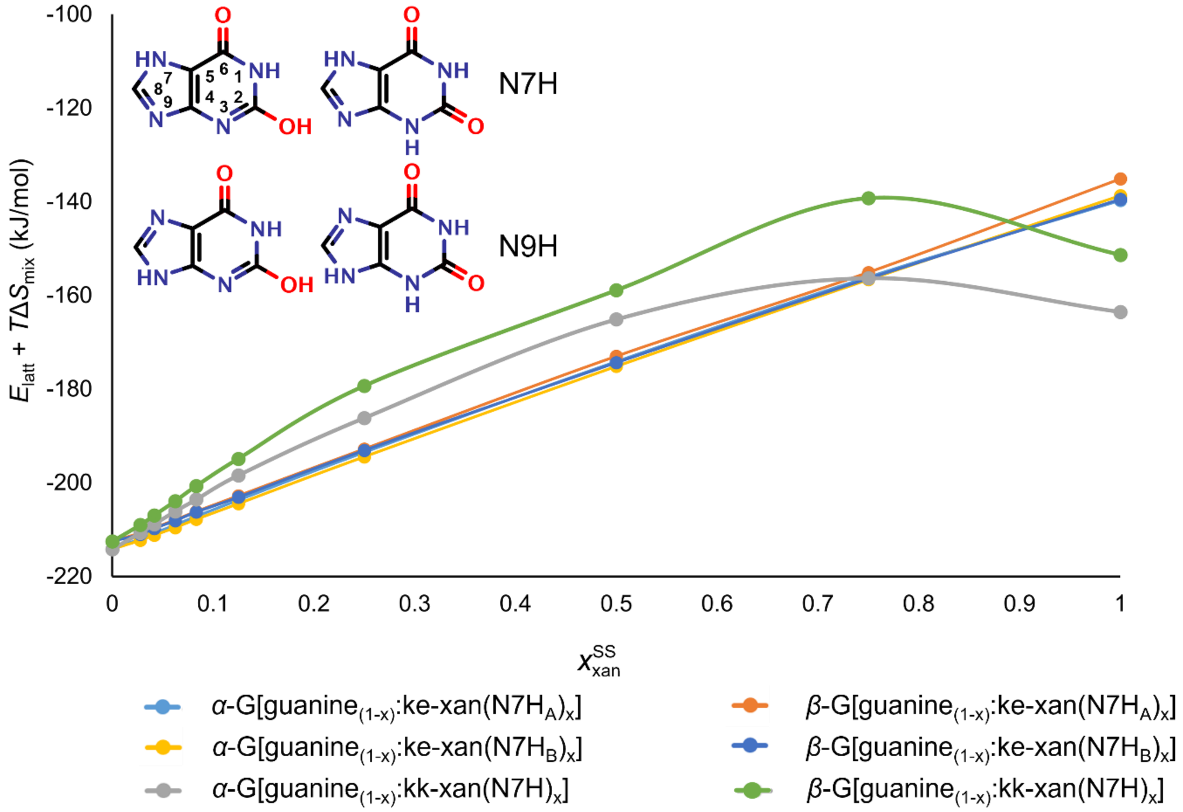

Figure S10. Lattice energy plots for all investigated tautomers of xanthine with added entropic corrections to account for solid solution formation (TS + MBD corrections at 0 K). The keto tautomer is shown to be much higher in energy than the enol tautomers at the x_{xan}^{SS} values observed experimentally ($x_{xan}^{SS} \leq 0.1$). The subscript A/B corresponds to different conformers originating from rotation of the enol O-H group (A = N9 acceptor and B = keto O6 acceptor, shown in Figure 5 in the main manuscript).

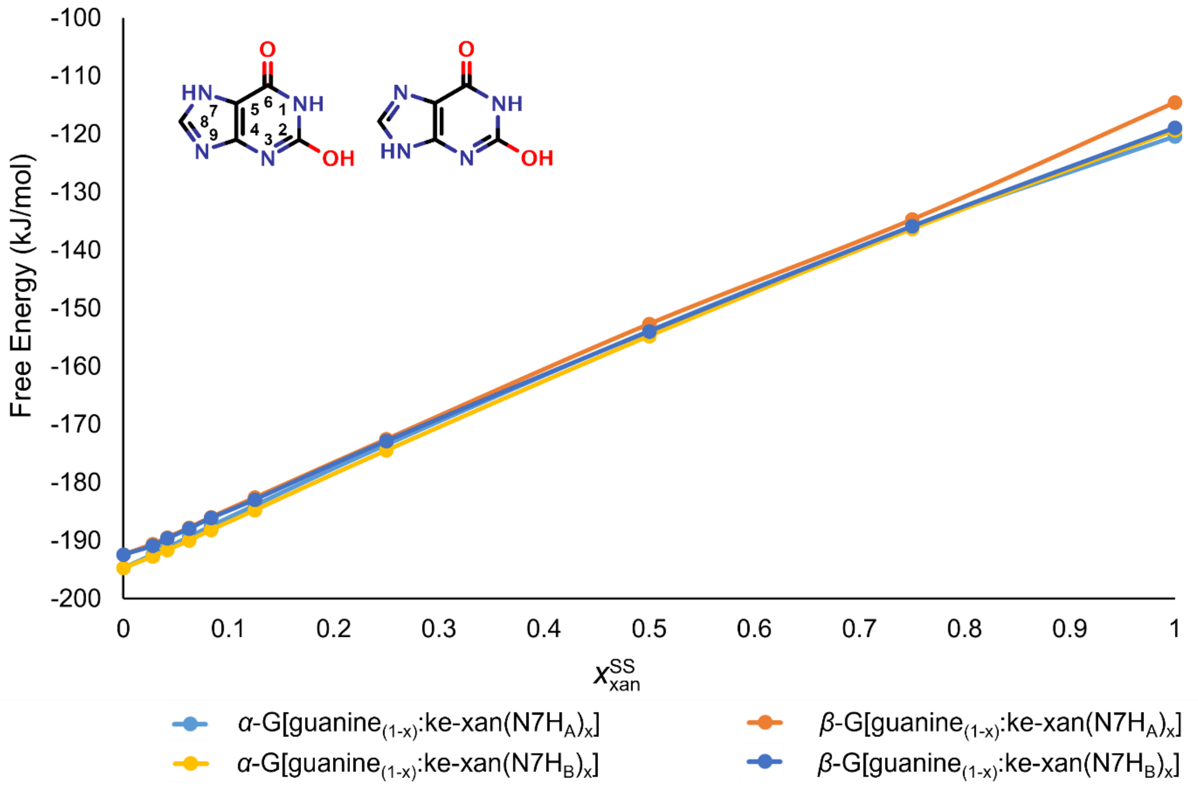

Figure S11. Free energy (at 300 K) calculations of solid solutions containing each conformer of the N7H enol tautomer for xan. The subscript A/B corresponds to different conformers originating from rotation of the enol O-H group (A = N9 acceptor and B = keto O6 acceptor, shown in Figure 5 in the main manuscript). All conformers are close in energy, but the B set are lower on average.

S2.1 Supersaturation and driving force calculation

A supersaturation ratio (σ) is given by:

$$\sigma = \frac{[\mathrm{A}]_{\mathrm{x}}}{[\mathrm{A}]_{\mathrm{Eq}}}$$

where [A] denotes the concentration (or activity) of species A, and the subscripts x and Eq denote an arbitrary concentration and equilibrium concentration (solubility), respectively.

The driving force towards crystallization ($\Delta\mu$ i.e. the difference in chemical potential between the liquid and crystalline state) is given by:

$$\Delta\mu = RT\ln(\sigma)$$

where R is the gas constant, T is the temperature in Kelvin and σ is the supersaturation ratio; positive values denote crystallization is favored over remaining in solution.

All compounds are in excess relative to their solubilities in water at pH 7, with the supersaturation of guanine reaching two orders of magnitude higher than xan and hpx (Figure S12). Converting supersaturation values to a thermodynamic driving force towards crystallization (Figure S13), the energy difference between the physical mixture and solid solutions is surpassed across the majority of

the $x_{\mathrm{hpx}}^{\mathrm{SS}}$ range. The system is thus in a non-equilibrium state, and the energy available is enough to overcome the kinetic barriers and crystallize as solid solutions.

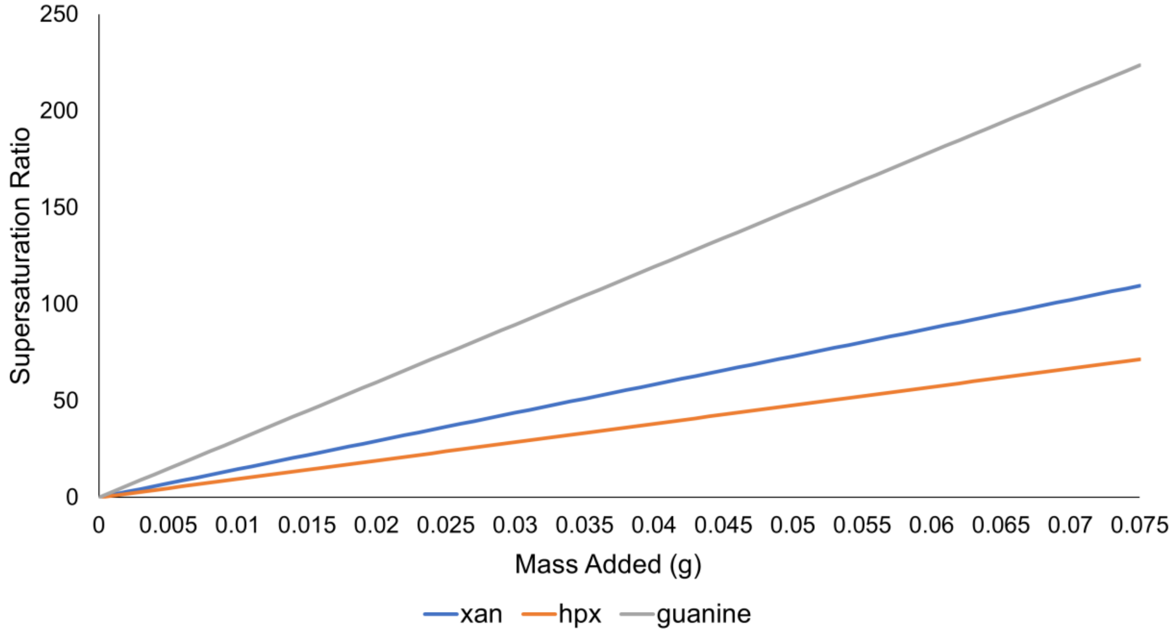

Figure S12. A plot of the supersaturation ratio for guanine, hypoxanthine and xanthine relative to their solubilities in water.

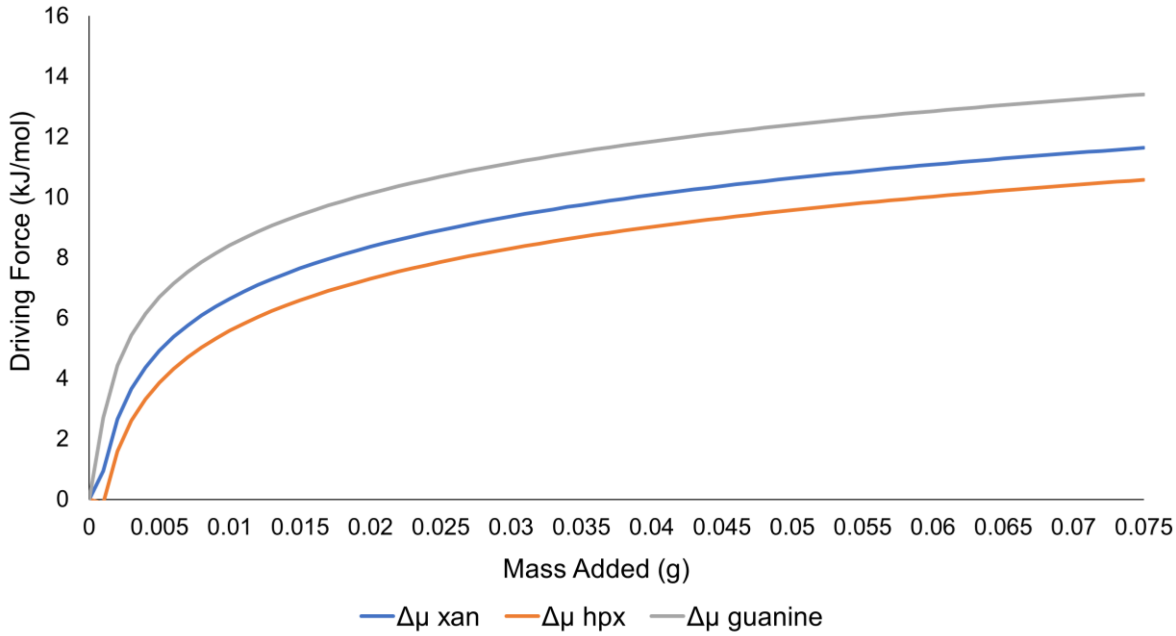

Figure S13. A plot of the thermodynamic driving force towards crystallization for guanine, hypoxanthine and xanthine relative to their solubilities in water.

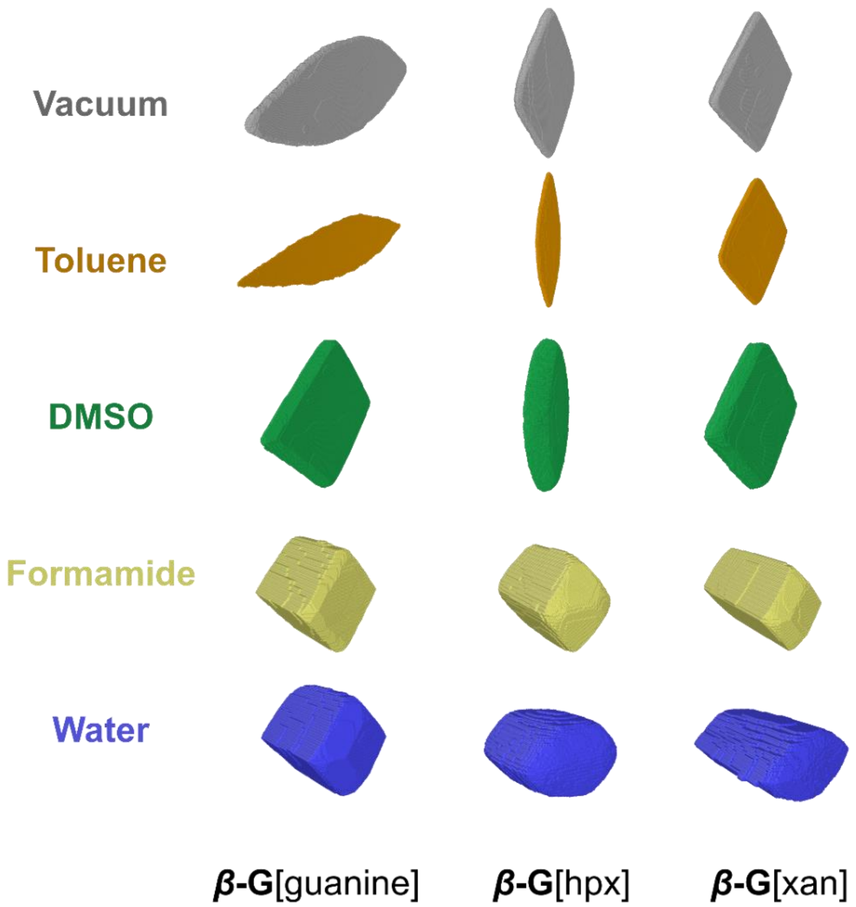

Figure S14. Morphologies for *β*-G[guanine], *β*-G[hpx] and *β*-G[xan] as output directly from **Crystal***Grower* and visualized with OVITO. Vacuum morphologies are shown in grey, toluene in orange, DMSO in green, formamide in yellow and water in blue. All crystals are displayed in the same coordinate space (offset from (100) face).

S2.2 Diketo-xan simulation details

No reported crystal structure exists for xan, but it is frequently shown to adopt the diketo tautomer. We simulated this tautomer by matching the 5-ring hydrogen position to guanine (N7H) also used to simulate hpx. The keto-enol tautomer was simulated and preferred after noting that this would eliminate steric hindrance and better maintain the hydrogen bonding network seen in the pure beta guanine structure (Figure 4a). The keto-enol tautomer was found to produce much lower lattice energies (~50 kJ/mol) than incorporating the diketo-tautomer (Figure S10). Due to this, only the keto-enol tautomer simulations were studied in detail for the main manuscript.

For the morphology simulations, all the host molecules in the unoptimized *β*-guanine structure (KEMDOW01 as obtained from the CSD) were replaced with diketo-xanthine. This structure required optimization with fixed unit cell parameters to separate molecules considered bonded due to steric clashing (Figure 4a). Then, this substituted structure was used as input for **Crystal***Grower*. These simulations resulted in dramatically different morphologies, with no {100} or {002} facets appearing

in the grown crystals and exhibited different sets of facets to the other simulated crystals. These changes are expected, as the crystal structure was altered substantially from pure β-guanine by the optimization process, with interactions pointing in different directions to the pure β-guanine structure, making it difficult to compare results between this system and others (Figure S15).

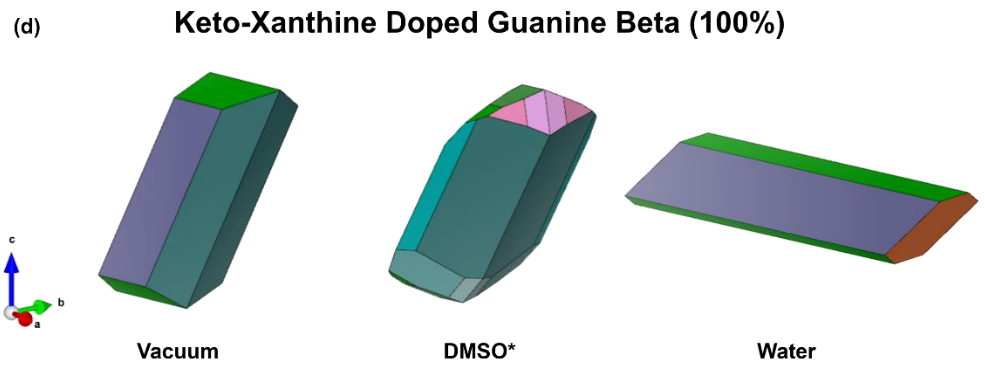

Figure S15. Schematics of simulated crystal morphologies in vacuum, DMSO and water for β-G[kk-xan]. Facet sets are colored using the same scheme for (a-c): green {100}, red {012}, purple {002}, blue {1$\bar{1}$2}, orange {1$\bar{1}$1}, and cyan {1$\bar{2}$0}. An alternative scheme was used for (d): purple {010}, green {011}, dark cyan {$\bar{1}$22}, pink {$\bar{2}$10}, and orange {104}, with similar colours denoting higher index faces in roughly the same orientation as the base colour. The asterisk denotes that the DMSO morphology was very rounded and difficult to distinguish facets. All crystals were grown with CrystalGrower and reconstructed using VESTA to display smooth facets.

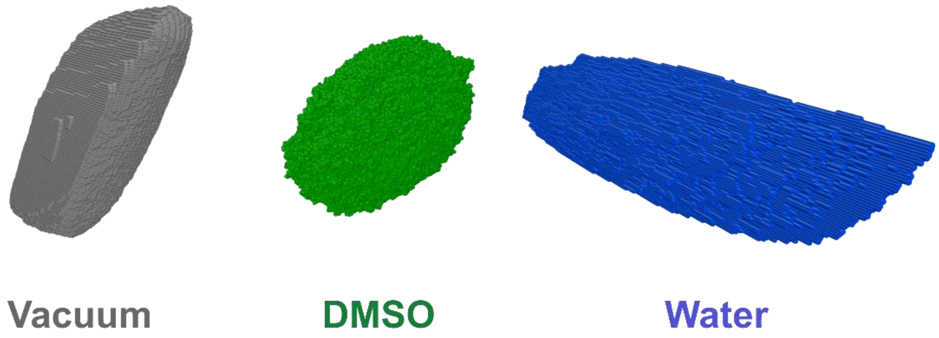

Figure S16. Morphologies for β-G[kk-xan] as output directly from **Crystal***Grower* and visualized with OVITO. Vacuum morphology is shown in grey, DMSO in green and water in blue.

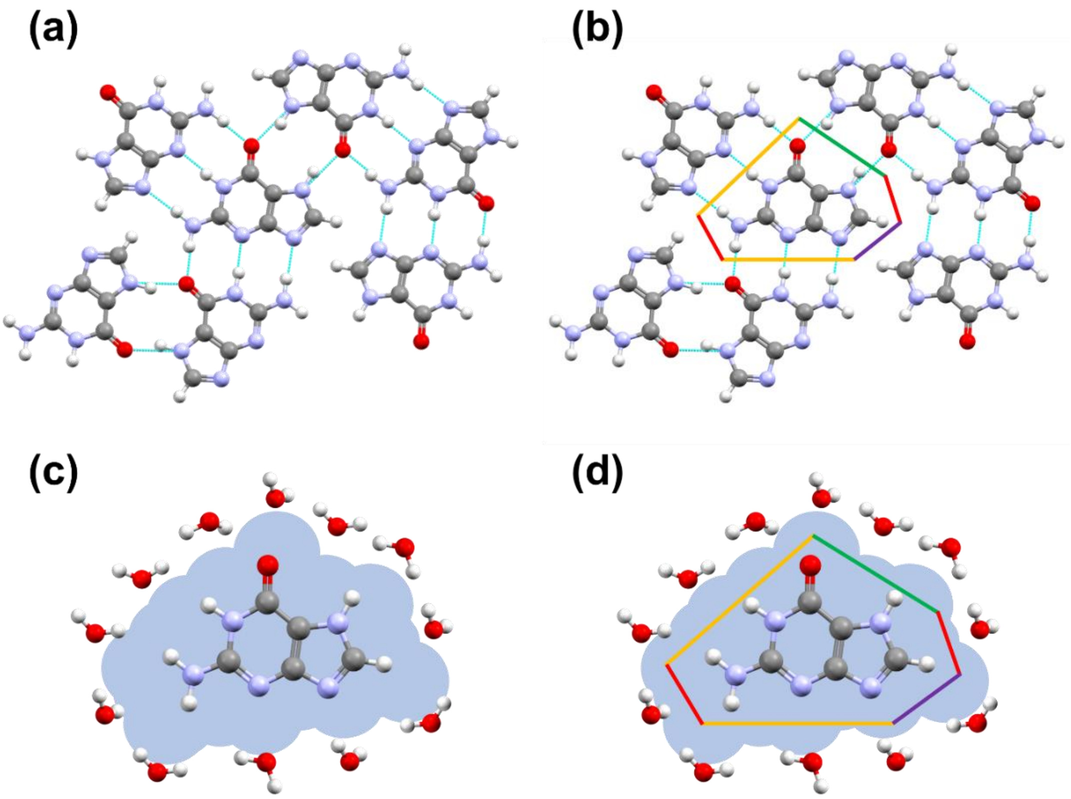

Figure S17. A simplified 2D schematic of the partitioning scheme used to calculate ΔG_{desolv} and $\Delta G_{cryst.}$ (a) a cross-section of the *bc* plane in the crystal structure of *β*-guanine, with interacting neighbor molecules added and hydrogen bonds shown by dashed blue lines. (b) a partitioned area highlighted around the crystal with sections corresponding to each neighbor interaction (colored by symmetry). (c) a graphic of an isolated guanine molecule in a hypothetical solvation field caused by water in the SMD model where no information about the crystal structure is retained (i.e. this will be the same, regardless of where the guanine molecule originates). (d) the same solvation field partitioned to match the neighbor interactions in the crystal structure, this will differ depending on the crystal structure the guanine originates from. The total energy of solvation is divided between the partitions corresponding to each neighbor interaction by area. In reality there are also π interactions running along the a-direction which will also have their own partition of the solvation field.

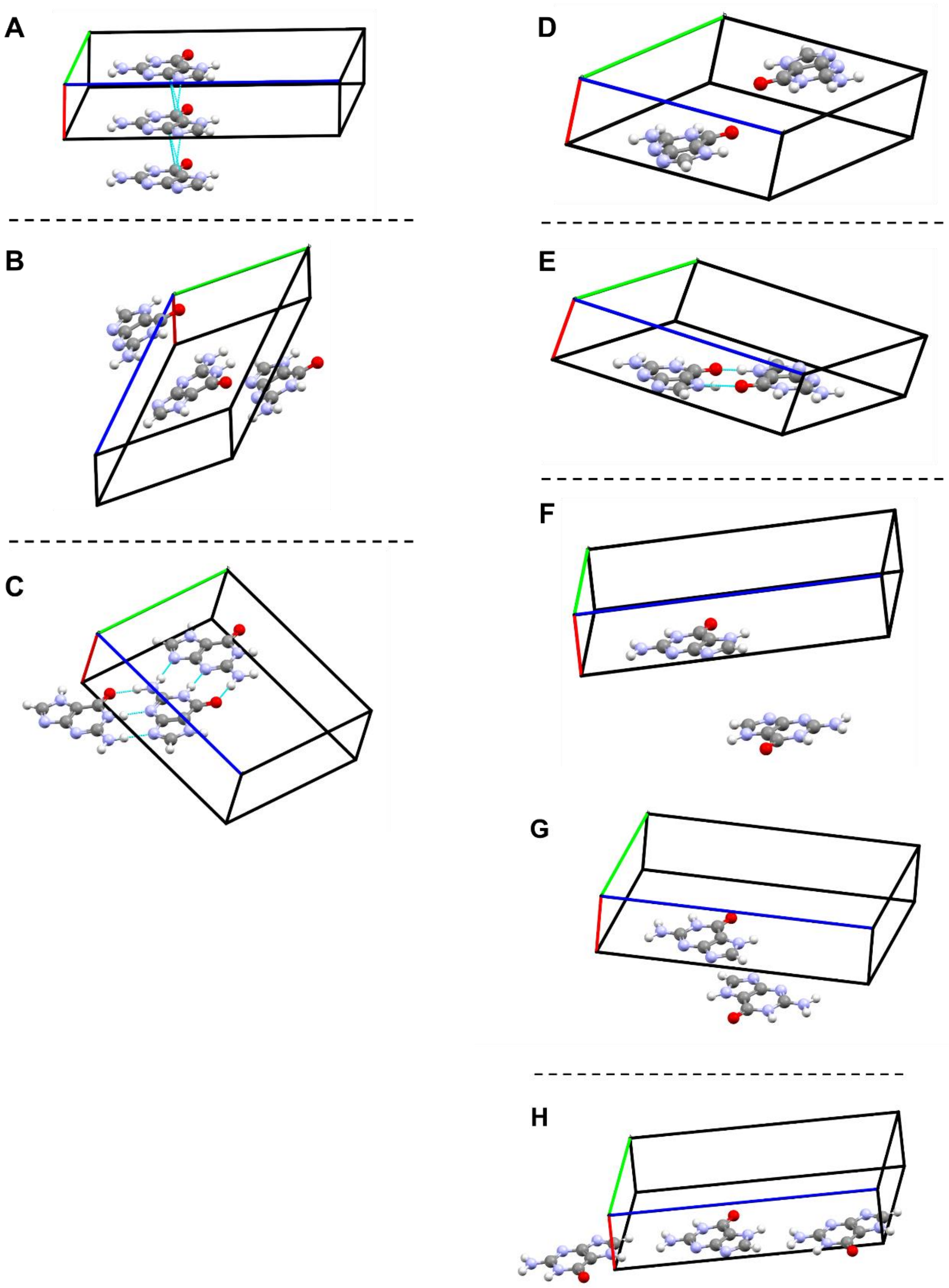

Figure S18. Images of the 8 (A-H) unique intermolecular interaction types considered during the morphology modelling of β-guanine and its isostructural analogues. The respective intermolecular interactions are kept constant between all structures, although their chemical makeup will change as functional groups are added, removed or changed. Centroid distances also change, as the centers of mass or geometry will change with atomic changes. Trimers indicate that interactions are equivalent on two sides due to symmetry caused by mirror planes, whereas dimers do not have symmetry-related copies.

Table S4. Details of interactions A-H in the pure *β*-guanine structure. Interactions are given short descriptions of the groups involved in the interaction, with reference to pure *β*-guanine and a shorthand name used for discussion in the manuscript. Neighbors which are related to neighbors involved in other interactions via π-stacking to adjacent layers in the *a*-direction are highlighted. Where chains of interactions are possible (i.e. symmetry related copies), an overall chain direction in the crystal structure is given. Pairs of interactions which contain related molecules could be considered as a "two-part" chain, where combining both interactions results in a chain of interactions running along a particular direction. These interactions, however, are governed by two free energies of crystallization, and can therefore be interrupted by altering said energies, unlike their symmetry-related counterparts.

Label	Description	Shorthand	Relation	Primary Chain Direction
A	π-stack	π-stack	N/A	[100] (a)
B	Diagonal aromatic (6-ring)	π3Hb	π-stack to interaction C	[110] (ab)
C	Triple H-bond	3Hbond	π-stack to interaction B	[010] (b)
D	Diagonal aromatic (5-ring)	π2Hb	π-stack to interaction E	[100] (a) when combined with E
E	Double H-bond	2Hbond	π-stack to interaction D	[100] (a) when combined with D
F	5-ring (out of plane)	π5r	π-stack to interaction G	[100] (a) when combined with G
G	5-ring (in plane)	5-ring	π-stack to interaction F	[100] (a) when combined with F
H	van der Waals chain (in plane)	Chain	Triple hydrogen bond to E	[001] (c)

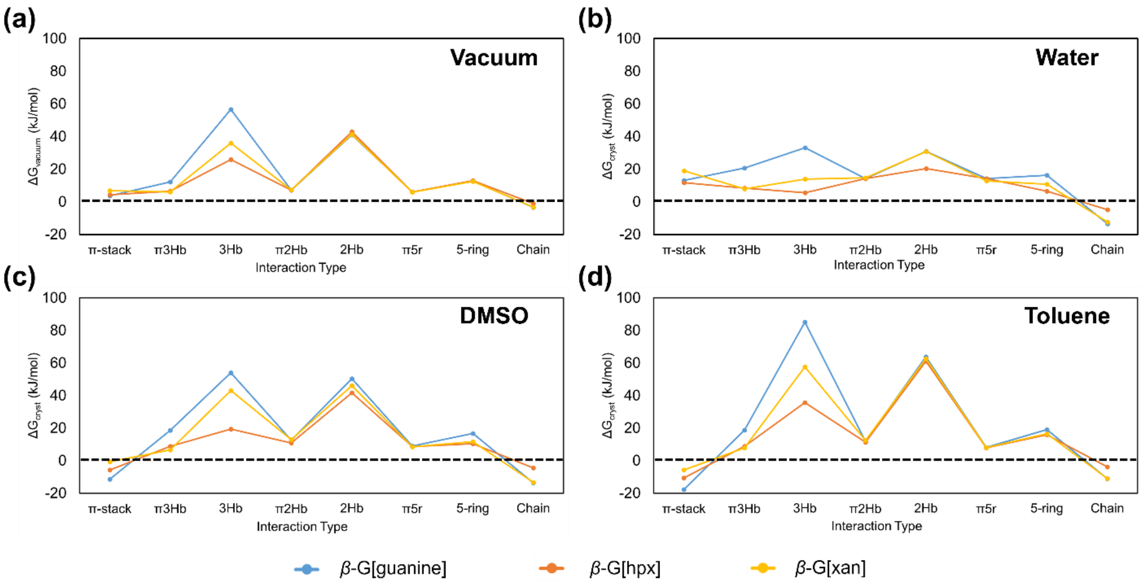

Figure S19. A set of plots showing the free energies of crystallization (ΔG_{cryst}) for each unique interaction in the β-guanine structure. (a) ΔG_{cryst} in the absence of solvent (ΔG_{vacuum}) which corresponds to the interaction energies in the solid phase alone (summing to E_{latt}), (b – d) ΔG_{cryst} in water, DMSO and toluene, respectively, where the cost of desolvation (ΔG_{desolv}) for interaction is subtracted from the interaction energy in the solid state (ΔG_{vacuum}) to result in ΔG_{cryst}. All interactions above the dashed line at 0 kJ / mol are favored to grow in the chosen solvent, while all those below are favored to dissolve. In the solid state, these would correspond to attractive and repulsive interactions, respectively.

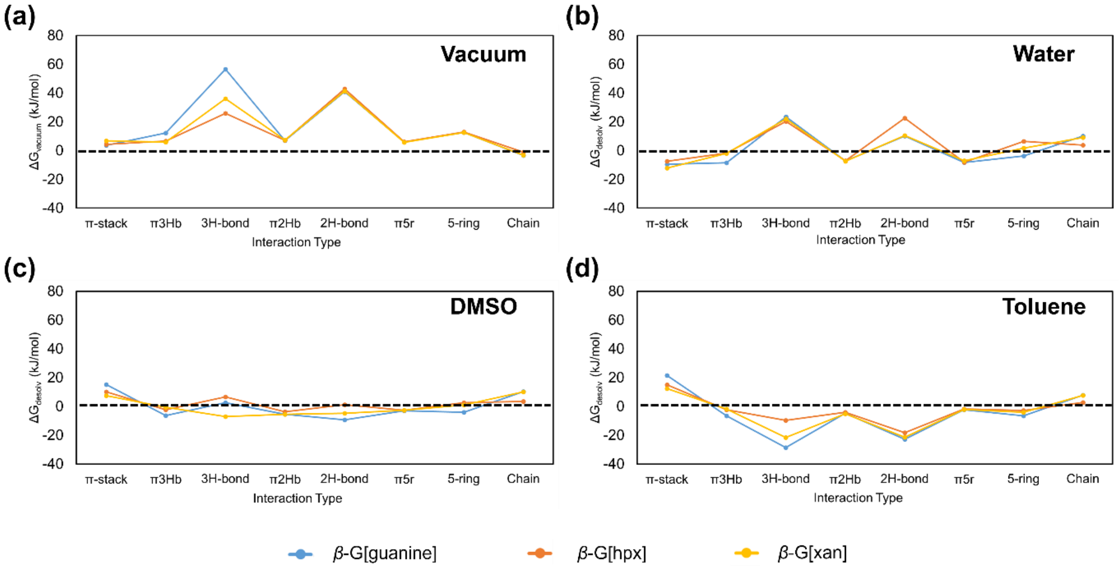

Figure S20. A set of plots showing the free energies of desolvation (ΔG_{desolv}) for each unique interaction in the β-guanine structure. (a) ΔG_{vacuum} to present the strength of each interaction in the solid state for reference, (b – d) ΔG_{desolv} in water, DMSO and toluene, respectively, where the solvation field has been portioned into areas corresponding to each interaction A-H. All interactions above the dashed line at 0 kJ / mol in (b – d) indicate a preference to remain in solution (i.e. they cost energy to desolvate) while those under the line indicate a preference to crystallize (i.e. they cost energy to solvate). ΔG_{desolv} follows expected behavior for solvent choice – e.g. the energy cost to desolvate hydrogen bonds (interactions C and E) in water are very high, whereas in toluene they are extremely low. Combination of plot (a) with (b – d) result in the ΔG_{cryst} plots.

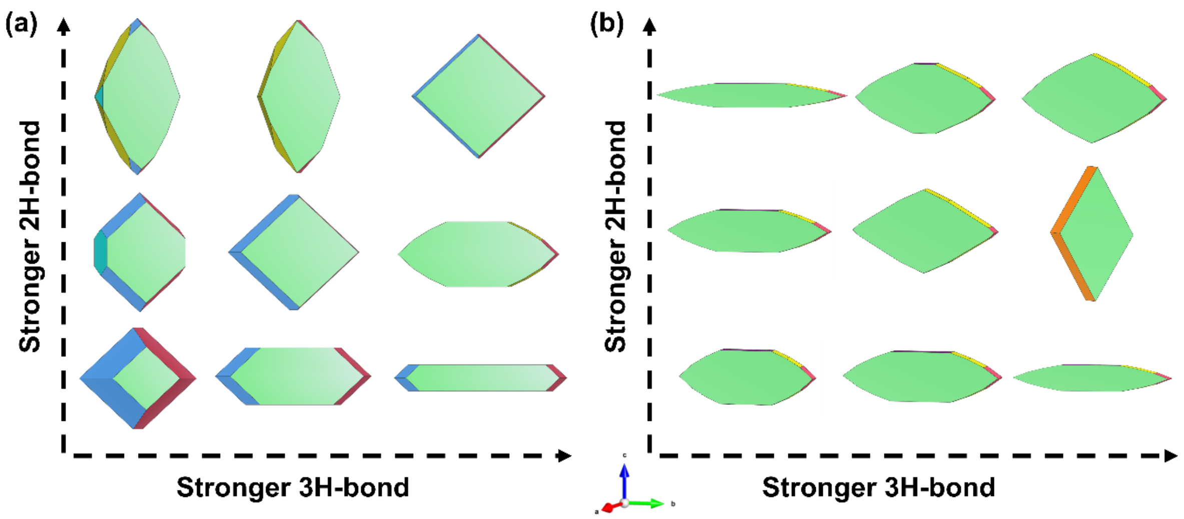

Figure S21. Morphology comparison for β-guanine varying the strength of 3H-bond and 2H-bond when (a) π interactions are left free to grow (b) the π interactions are blocked by assignment of an energy penalty (-5 kcal/mol) to mimic the effect of a capping agent. All capped morphologies are much flatter and platelike than the uncapped morphology.

S3. Supplementary movies

Supplementary Movies SM1-SM2. Simulations of *β*-G[guanine$_{1-x}$:hpx$_x$] and *β*-G[guanine$_{1-x}$:xan$_x$] solid solutions in water, with 3H-bond strength varied between guanine and guest to control guest incorporation. Simulations were constructed by overlaying the pure *β*-guanine and *β*-G[hpx] or *β*-G[xan] structures in the same crystal unit cell. Effectively, this creates a net where nodes represent the centroids for either guanine, hpx or xan, with the edges representing the interactions between said molecules. All connections were allowed, aside from between guanine and hpx or xan which would occupy the same crystallographic site (i.e. no clashes allowed). ΔG_{cryst} values for guanine:guanine and guest:guest interactions were used from the OCC computations shown in Figure S17b, and guanine:guest interactions were taken as an average between the two extremes. 3H-bond strengths between guest and guanine were varied from 0 – 20 kcal/mol (i.e. moving from no guest:host interaction to favoring guest:host interaction). The movies show that solid solution morphologies tend to lie between the two extremes of pure guanine in water (symmetric rod) and the *β*-G[hpx] or *β*-G[xan] morphologies, justifying the focus of *β*-G[hpx] and *β*-G[xan] for discussion in the manuscript.

Supplementary Movies SM3-SM5. Timelapse movies of pure *β*-guanine, *β*-G[hpx] and *β*-G[xan] grown in water. ΔG_{cryst} values used are identical to those shown in Figure S20b. 100 simulation frames were output across 1 million growth / dissolution iterations (10,000 iterations per frame), with the first 50 frames at high supersaturation (100 kcal/mol) then dropping to equilibrium for frames 50-60 and held at equilibrium for the remaining 40 frames. The final frame of each simulation was used as the basis for the final morphology for the compound in water.
